# Shortcuts to Thermodynamic Computing: The Cost of Fast and Faithful Information Processing

**DOI:** 10.1007/s10955-022-02871-0

**Published:** 2022-03-28

**Authors:** Alexander B. Boyd, Ayoti Patra, Christopher Jarzynski, James P. Crutchfield

**Affiliations:** 1 Complexity Sciences Center and Physics Department, University of California at Davis, One Shields Avenue, Davis, CA 95616 USA; 2 Department of Physics, University of Maryland, College Park, MD 20742 USA; 3 Department of Chemistry and Biochemistry, University of Maryland, College Park, MD 20742 USA; 4 Institute for Physical Science and Technology, University of Maryland, College Park, MD 20742 USA

**Keywords:** Thermodynamic computing, Optimal transport, Landauer’s bound, Entropy

## Abstract

Landauer’s Principle states that the energy cost of information processing must exceed the product of the temperature, Boltzmann’s constant, and the change in Shannon entropy of the information-bearing degrees of freedom. However, this lower bound is achievable only for quasistatic, near-equilibrium computations—that is, only over infinite time. In practice, information processing takes place in finite time, resulting in dissipation and potentially unreliable logical outcomes. For overdamped Langevin dynamics, we show that counterdiabatic potentials can be crafted to guide systems rapidly and accurately along desired computational paths, providing shortcuts that allow for the precise design of finite-time computations. Such shortcuts require additional work, beyond Landauer’s bound, that is irretrievably dissipated into the environment. We show that this dissipated work is proportional to the computation rate as well as the square of the information-storing system’s length scale. As a paradigmatic example, we design shortcuts to create, erase, and transfer a bit of information metastably stored in a double-well potential. Though dissipated work generally increases with operation fidelity, we show that it is possible to compute with perfect fidelity in finite time with finite work. We also show that the robustness of information storage affects an operation’s energetic cost—specifically, the dissipated work scales as the information lifetime of the bistable system. Our analysis exposes a rich and nuanced relationship between work, speed, size of the information-bearing degrees of freedom, storage robustness, and the difference between initial and final informational statistics.

## Introduction

Information processing requires work. For example, no less than $k_\text {B}T \ln 2$ of work must be supplied in order to erase a single bit of information at temperature *T* [1]. More generally, Landauer’s Principle bounds the work investment by the change in the memory’s Shannon entropy [2]:

1 $$\begin{aligned} \langle W \rangle \ge k_\text {B}T \ln 2 \left( {\text {H}}[Y_0] - {\text {H}}[Y_\tau ] \right) . \end{aligned}$$

Here, $Y_0$ and $Y_\tau$ are random variables describing initial and final memory states with equal free energies, and ${\text {H}}[Y]\,{=}-\sum _{y} \Pr (Y{=}y) \log _2 \Pr (Y{=}y)$ denotes the uncertainty in bits of a random variable *Y*.

Mathematically, information processing is described by a communication channel [3] that maps an initial distribution $\Pr (Y_0)$ to a final distribution $\Pr (Y_\tau )$. Physically, a memory is realized by a system whose thermodynamically-metastable states encode logical states $\{y\}$. The simplest example is a Brownian particle in a double-well potential, with two deep wells representing the $y=0$ and $y=1$ states of a single bit of information. More generally, the collection of all possible memory states ${\mathcal {Y}}=\{y\}$ represents a mesoscopic coarse-graining of the space of explicit physical microstates ${\mathcal {X}}=\{x\}$ of the memory device. Information processing is implemented by varying the system’s energy landscape so as to drive the flow of probability between memory states in a controlled fashion, to achieve a desired computation.

A computation can be implemented to achieve the Landauer bound, Eq. (1), by varying the energy landscape infinitely slowly, so that the system remains in metastable equilibrium from beginning to end [4, 5]. Such quasistatic computations, however, take infinitely long to implement. For computations performed in finite time the underlying physical system is driven out of equilibrium, resulting in the irretrievable dissipation of energy into thermal surroundings. This dissipation has been explored in the near-equilibrium linear-response regime, showing intriguing dependence on the rate of computation [6–11], length scale [11], and on the distance between the initial and final distribution [7]. Many of these results employ the tools of geometric thermodynamic control to minimize the dissipation of near-equilibrium systems [6–9].

Here, we address the separate design problem of *implementing a computation rapidly and faithfully, allowing the system to be far-from-equilibrium*. That is, we show how to design protocols that vary a system’s energy landscape, so as to produce a desired computation in a given time interval, no matter how short its duration. In effect, we place a premium on speed of computation rather than on energy efficiency. That said, we then proceed to analyze the energetic costs of rapid computation, reproducing many of the dependencies observed for linear response. However, the results we obtain are not limited to that regime—they remain valid even when the system is driven far-from-equilibrium during information processing.

To achieve rapid and precisely-controlled information processing, we use recently developed tools from the field of *shortcuts to adiabaticity* [12]. Specifically, we adapt the methods of counterdiabatic control of classical overdamped systems [13], originally inspired by pioneering experiments on the engineered swift equilibration of a Brownian particle [14], to the task of general information processing. Though the rate dependence of dissipation in counterdiabatic protocols and in other far-from-equilibrium thermodynamic control has been explored previously [15–23] we find further dependencies by applying the techniques to metastable information processing. While restricting to metastable distributions prevents fully optimal control, it respects the inherent information storage capacity of the physical system and leads to intriguing relationships between information storage robustness, time, space, and the type of computation. Within the framework of metastable computing, we demonstrate a wide range of computational design.

For concreteness, we show how to apply counterdiabatic control to create, erase, and transfer a single bit of information rapidly and accurately. That said, our approach addresses general information processing, which we illustrate by analyzing thermodynamic controls for creating and transferring bits.

To embed the memory states ${\mathcal {Y}}$ physically, we consider a one-dimensional position space ${\mathcal {X}}$ governed by overdamped Fokker–Planck dynamics. The energy landscape at the beginning and end of the protocol is the double-well potential shown in Fig. 2, with a barrier sufficiently high to prevent the leakage of probability between the two wells. Thus, the landscape provides a means of storing information in metastable mesoscopic states. As we will show, counterdiabatic control of the potential can be used to drive any initial distribution over the memory states to any desired final distribution in finite time—in fact, arbitrarily rapidly. Mirroring results in geometric control, we show that the work required to perform this counterdiabatic process decomposes into a change in free energy, which captures Landauer’s change in state space cost, plus an additional contribution that scales as the rate of computation and the square of the length scale of the information-storing potential [7]. This additional work is proportional to the global entropy production and so quantifies thermodynamic inefficiency.

Our approach reveals additional trade-offs beyond that between computation rate, length scale, and thermodynamic efficiency. We show that dissipation also increases with the difference between initial and final bit distributions of the computation and with the robustness of information storage. In this way, we give a more complete picture of *metastable* information processing beyond Landauer’s bound. Rather than a tradeoff between information and energy, more complex tradeoffs are revealed between information, energy, statistical bit-bias difference, speed, size of the memory states, and information robustness. This is accomplished within a single, unified framework that, in many respects, is markedly more tractable than previous approaches.

## Thermodynamic Computing

What is physical computing? At the outset, information must be encoded in collections of microscopic states ${\mathcal {X}}$ of a physical system. Let ${\mathcal {Y}}$ denote these information-containing microstate groups—the accessible *memory states* [24, 25]. By manipulating the physical system, a microstate collection evolves, transforming the information it contains. Generally, an information processor has only partial control over the underlying microstates of its physical implementation, because the energy landscape has limited tunable parameters. Similarly, information processors only have partial knowledge of the microstates, which are coarse-grained into observable macrostates. We now consider how such information processing can be modeled by stochastic dynamics governed by a controlled potential.

### Memory States and Symbolic Dynamics

There are many ways to form memory states out of physical microstates. Here, we choose a framework for information erasure and general information processing in which the physical degrees of freedom ${\mathcal {X}}$ participate in metastable equilibria. Each metastable equilibrium is a microstate distribution that corresponds to a memory state $y \in {\mathcal {Y}}$. For example, we can have memory states ${\mathcal {Y}}=\{0,1\}$, such that they are stable for intermediate, if not asymptotically long, time scales. The coarse-graining $c: {\mathcal {X}} \rightarrow {\mathcal {Y}}$ of physical states to form the informational states specifies the memory alphabet ${\mathcal {Y}}= \{c(x) |x \in {\mathcal {X}}\}$. This translates a distribution $\Pr (X_t)$ over physical microstates $x \in {\mathcal {X}}$ to a distribution $\Pr (Y_t)$ over informational states $y \in {\mathcal {Y}}$. In this way, controlling a physical system determines not only its raw physical dynamics, but also the *symbolic dynamics* of the informational states.^1^

We use random variable notation, $\Pr (X_t)= \{ (x,\Pr (X_t=x)), x \in {\mathcal {X}}\}$, common in symbolic dynamics [27], rather than $\rho (x,t)$, which is more standard in stochastic thermodynamics, due to its specificity and flexibility. The probability of being in microstate *x* at time *t* is expressible in both notations $\Pr (X_t=x)=\rho (x,t)$, but the random variable notation works with many different distributions over the same microstate space ${\mathcal {X}}$. And so, rather than specify many different probability functions, we specify their random variables. Other advantages of this choice is that it readily expresses joint probabilities, such as $\Pr (X_t=x,X_{t+\tau }=x')$, and entropies:

2 $$\begin{aligned} {\text {H}}[X_t]\,{=}-\sum _{x \in {\mathcal {X}}} \Pr (X_t{=}x) \log _2 \Pr (X_t{=}x) . \end{aligned}$$

While not all of the potential functionality is used in the following, a number of recent results in stochastic thermodynamics have used the power of this notation to express stochastic processes to great effect [4, 28].

### Overdamped Fokker–Planck Dynamics

The first challenge of thermodynamic computing is to control a system’s Hamiltonian over the physical degrees of freedom ${\mathcal {X}}$ such that the induced microstate distribution $\Pr (X_t)$ at time *t* matches a desired distribution $\Pr (X^d_t)$, where $X_t$ and $X^d_t$ are the random variables for the actual physical distribution and desired physical distribution, respectively, at time *t*, each realizing states $x \in {\mathcal {X}}$. The second challenge, which we come to later, is to associate the microstate distributions with mesostate distributions that support the desired information-storing and -processing.

We consider a Hamiltonian controlled via a potential energy landscape *V*(*x*, *t*) over the time interval $t \in (0,\tau )$, where $x\in {\mathcal {X}}$. We will demonstrate that one can exactly guide an overdamped Fokker–Planck dynamics in one dimension along the desired time sequence of distributions $\Pr (X^d_t=x)$, resulting in a powerful tool for thermodynamic control and information processing.

In fact, overdamped stochastic systems are a promising and now common framework for elementary thermodynamic information processing [29, 30]. With a single physical degree of freedom ${\mathcal {X}}={\mathbb {R}}$, one information processing task is to change the initial distribution to a final distribution in finite time. The actual microstate distribution $\Pr (X_t)$ obeys the Fokker–Planck equation:

3 $$\begin{aligned} \frac{\partial \Pr (X_t=x)}{\partial t}&= \mu \frac{\partial }{\partial x} \left( \Pr (X_t=x)\frac{\partial V(x,t)}{\partial x}\right) +\mu k_\text {B}T \frac{\partial ^2 \Pr (X_t=x)}{\partial x^2} , \end{aligned}$$

where *V*(*x*, *t*) is the potential energy landscape at time *t*, *T* is the temperature of the thermal environment, and $\mu$ is the inverse friction coefficient.

We can use these stochastic dynamics to design a computation by evolving particle distributions. It is useful to recognize that, for appropriately bounded energy landscapes *V*(*x*, *t*), the stationary distribution for the Fokker–Planck equation is a normalized Boltzmann equilibrium distribution, if the potential is held fixed at time *t*:

4 $$\begin{aligned} \Pr (X^\text {eq}_t=x) = \frac{e^{-V(x,t)/k_B T}}{Z(t)}, \end{aligned}$$

with partition function $Z(t)\equiv \sum _{x}e^{-V(x,t)/k_B T}$. That is, substituting into the righthand side of Eq. (3) yields:

$$\begin{aligned} \frac{\partial \Pr (X^\text {eq}_t=x)}{\partial t} = 0. \end{aligned}$$

We can therefore use this equilibrium distribution as a guidepost for designing thermodynamic computations as described in greater detail in Sec. 3.

## Work Production During Counterdiabatic Protocols

Next, we identify how the evolution of the physical distribution yields useful changes in memory states that robustly store a computation’s result. We break the development into two parts.

This section considers counterdiabatic Hamiltonian control of the physical states $x \in {\mathcal {X}}$ such that they follow specified distributions $\Pr (X^d_t)$ over the time interval $t \in (0, \tau )$ [13]. The goal of such counterdiabatic driving is to fast-forward a mapping between an initial and final equilibrium distribution—a process that would take infinitely long if we followed the equilibrium distribution for the entire protocol. For the resulting finite-time protocol, we determine the work production and show that it increases with both the size of the memory states and the speed of operation, if the overall computational task is fixed. This holds for any counterdiabatically-controlled computation.

The subsequent section addresses the particular computational task of information erasure in a bistable potential well. Such computational processes require metastable storage of information that, in turn, rely on potentially nonequilibrium initial and final distributions—in this way, extending the framework of counterdiabatic computation. While the analytical and numerical results there do not explicitly generalize to other computational tasks, they introduce general relationships between dissipated work, information storage robustness, and computation fidelity that hold broadly.

### Inverse Problem for Thermodynamic Control

For a specified potential *V*(*x*, *t*), the Fokker–Planck equation Eq. (3) evolves an initial distribution $\Pr (X_0)$ to a density $\Pr (X_t)$ at any later time in the control interval $t \in (0,\tau )$. Together, the probability density and potential determine the average energy expended as work $\langle W \rangle$ by the protocol on the physical system [25]:

5 $$\begin{aligned} \langle W \rangle = \int _{0}^\tau dt \int _{-\infty }^{\infty }dx \Pr (X_t=x) \partial _t V(x,t) . \end{aligned}$$

What if, rather than starting with an initial distribution and control protocol, we are given a desired trajectory of probability distributions $\Pr (X^d_t)$ over some time interval $t \in [0, \tau ]$—a *distribution trajectory*—and are tasked to determine the control protocol that yields the trajectory? This challenge—the *inverse problem* of reconstructing dynamical equations of motion from distributions over trajectories—falls within purview of state-space reconstruction [31, 32] and computational mechanics [33] which provide principled approaches for inferring generators of observed time series. Broadly speaking, our challenge here is to reconstruct dynamical equations of motion for evolving distributions that perform computations and, then, to show how the work cost relates to the computation’s effectiveness. The setting here is both more constrained and more challenging than state-space reconstruction.

Generally, as with most inverse problems, determining the control protocol from a distribution trajectory does not lead to a unique solution. Many different dynamical systems can generate the same observed distributions [34]. Moreover, these inverse problems can be so challenging that machine learning represents one of the few promising candidates for effective solutions [35].

In this light, counterdiabatic techniques provide a *constructive* method for determining control protocols *V*(*x*, *t*) that produce the desired distribution trajectory $\Pr (X_t^d)$. This strategy has been applied to one-dimensional systems with a ring topology [36], or rate equations with arbitrarily many cycles in the topology [37], to determine the methods and corresponding cost of quickly transforming systems. These solutions are still not unique, since cycles in the topology lead to various possible control trajectories and corresponding energy costs for a particular computation.

However, for the specific case of overdamped Fokker–Planck dynamics in a single dimension ${\mathcal {X}}= {\mathbb {R}}$, Appendix A shows that the distribution trajectory $\Pr (X^d_t)$ uniquely determines the control protocol *V*(*x*, *t*) up to a baseline energy *E*(*t*) that is constant in position and so adds no force. This baseline energy can be any spatially uniform energy function and it will preserve the stochastic dynamics of the Brownian particles when added to *V*(*x*, *t*). Moreover, if $\Pr (X^d_t)$ characterizes our desired computation then, up to a readily-recovered change in baseline energy $E(\tau )-E(0)$, the work is uniquely determined for that computation. Thus, by designing a single protocol that guides the system along the desired distribution trajectory, we find both the unique protocol and the unique work investment required for that trajectory.

**Fig. 1:**
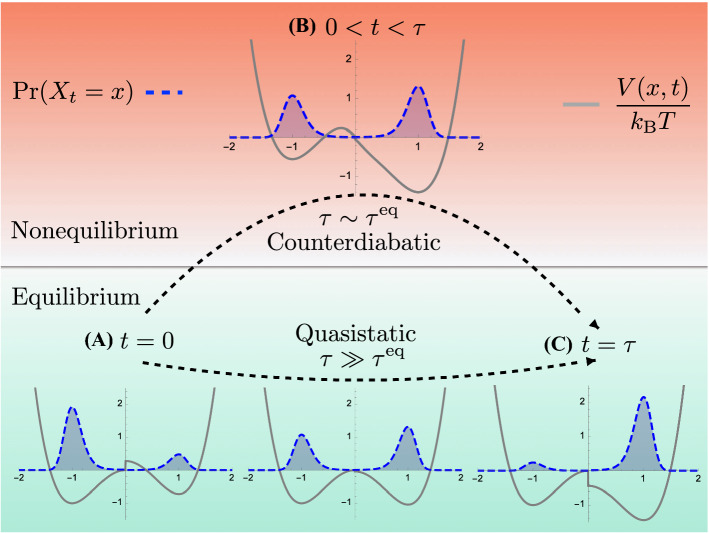
Counterdiabatic control of the energy landscape *V*(*x*, *t*) (solid gray curve) at times along the interval $t \in [0,\tau ]$ guides the probability distribution $\Pr (X_t=x)$ (dashed blue curve) along a desired trajectory $\Pr (X^d_t=x)$ in finite time $\tau$. The system starts in equilibrium in stage (A) and ends in equilibrium at stage (C), meaning that $V(x,0)=V^Q(x,0)$ and $V(x,\tau )=V^Q(x,\tau )$ are the quasistatic potentials for the initial and final distributions, respectively. However, at intermediate times, in stage (B), the necessary control protocol *V*(*x*, *t*) required to guide the system along the desired distribution changes as we change the speed of the protocol. If the timescale of equilibration is relatively very small $\tau ^\text {eq} \ll \tau$, then the control protocol must be in equilibrium with the desired distribution, such that the control potential is described by the quasistatic potential $V(x,t)=V^Q(x,t)$, as shown in the lower half of stage (B). Otherwise, an additional counterdiabatic term $V^{CD}(x,t)$ is added to the potential energy, which pushes the system out of equilibrium, as shown in the upper half of stage (B)

When $\tau$ is very large, a control protocol can be determined by assuming the system remains approximately in equilibrium at all times: $\Pr (X_t=x) \approx \Pr (X^\text {eq}_t=x)$. This follows from the system’s natural relaxation timescale $\tau ^\text {eq}$ that determines how long it takes to reach equilibrium, if the energy landscape is held fixed. When $\tau \gg \tau ^\text {eq}$, the changes in the energy landscape are so slow that the control protocol is *quasistatic* (adiabatic) and determined from the *quasistatic potential*:

6 $$\begin{aligned} V^Q(x,t) \equiv F^\text {eq}(t)-k_\text {B}T \ln \Pr (X^d_t=x) , \end{aligned}$$

where the equilibrium free energy:

7 $$\begin{aligned} F^\text {eq}(t)&= -k_\text {B}T \ln Z(t) \nonumber \\&=-k_\text {B}T \ln \int _{-\infty }^{\infty } dx e^{-V^{Q}(x,t)/k_\text {B}T} \end{aligned}$$

is the baseline energy *E*(*t*). Note that $\Pr (X^d_t)$ is the equilibrium distribution corresponding to $V^Q(x,t)$, see Eq. (4). In the large-$\tau$ case, the system follows this equilibrium distribution, as shown in Fig. 1, and the quasistatic protocol provides the unique solution to our control problem. Moreover, the work invested is the change in equilibrium free energy:

8 $$\begin{aligned} \langle W^Q \rangle = \Delta F^\text {eq} . \end{aligned}$$

If $\tau$ is *not* much larger than $\tau ^\mathrm{eq}$, however, then evolution under the quasistatic potential $V^Q(x,t)$, defined by Eq. (6), does not drive the system along the desired trajectory distribution $\Pr (X_t^d)$. Rather, the actual distribution $\Pr (X_t)$ deviates from the desired distribution as the system is pushed away from equilibrium.

Fortunately, recent results [13] describe how to construct a *counterdiabatic protocol* that achieves the desired evolution, $\Pr (X_t)=\Pr (X^d_t)$, for all $t \in (0,\tau )$. In this approach the overdamped system evolves under a potential:

9 $$\begin{aligned} V(x,t)=V^Q(x,t)+V^{CD}(x,t) , \end{aligned}$$

consisting of both the quasistatic term $V^Q(x,t)$ and a *counterdiabatic potential* $V^{CD}(x,t)$. The latter is constructed to guarantee that the actual distribution tracks the desired distribution, $\Pr (X_t) = \Pr (X^d_t)$, as illustrated in Fig. 1.

By Eq. (6) $\Pr (X^d_t)$ is the equilibrium distribution corresponding to the quasistatic potential $V^Q(x,t)$, but it is not the equilibrium distribution corresponding to the total potential *V*(*x*, *t*) given by Eq. (9). Thus, when the system evolves under the counterdiabatic protocol, it is out of equilibrium with respect to the instantaneous potential *V*(*x*, *t*) at intermediate times $t \in (0,\tau )$. However, to ensure that the system starts and ends in equilibrium, we choose $\Pr (X^d_t)$ such that $\partial _t \Pr (X^d_t)$ vanishes at the protocol’s start and end. This way the counterdiabatic potential vanishes at the endpoints of the protocol: $V^{CD}(x,t \in \{0,\tau \})=0$. And so, the potential energy becomes the quasistatic potential at the start $V(x,t=0)=V^Q(x,t=0)$ and end $V(x,t=\tau )=V^Q(x,t=\tau )$, as shown in Fig. 1.

### Counterdiabatic Control of Stochastic Systems

Reference [13] showed that the counterdiabatic potential $V^{CD}(x,t)$ is constructed from the desired distribution $\Pr (X^d_t)$ by integrating a velocity *flow field* *v*(*x*, *t*), defined shortly:

10 $$\begin{aligned} V^{CD}(x,t)=-\frac{1}{\mu } \int _{0}^xv(x',t)dx' . \end{aligned}$$

The lower limit of integration is set to 0 for convenience. In fact, it may take any value, as the physics is unchanged by the addition of an arbitrary function *f*(*t*) to the potential. For instance, Eq. (10) admits the solution for the velocity field described in Ref. [38]. Most generally, the velocity flow field:

$$\begin{aligned} v(x,t)&= \frac{\partial x}{\partial t} \biggr |_{C=\text {const}} \\&= - \frac{\partial _t C}{\partial _x C} \end{aligned}$$

is the velocity of constant values of the cumulative distribution function:

$$\begin{aligned} C(x,t)=\int _{-\infty }^x \Pr (X^d_t=x') dx' . \end{aligned}$$

Combining results, we have, explicitly:

11 $$\begin{aligned} V^{CD}(x,t)=\frac{1}{\mu } \int _{0}^x \int _{-\infty }^{x'} \frac{\partial _t\Pr (X^d_t=x'')}{\Pr (X^d_t=x')} dx'' dx' , \end{aligned}$$

for $t \in (0,\tau )$. For $t \notin (0,\tau )$ we set $V^{CD}(x,t)=0$, hence $V(x,t) = V^Q(x,t)$ outside of the control interval. As a result, the system begins in the equilibrium distribution at $t=0$ and it ends (and subsequently remains) in equilibrium at $t\ge \tau$.

Since the potential energy *V*(*x*, *t*) consists of quasistatic and counterdiabatic terms, we can similarly decompose the work in Eq. (5) into two contributions:

12 $$\begin{aligned} \langle W \rangle&= \int _{0}^\tau dt \int _{-\infty }^\infty dx \Pr (X_{t}=x) \partial _t V^Q(x,t) + \int _{0}^\tau dt \int _{-\infty }^\infty dx \Pr (X_{t}=x) \partial _t V^{CD}(x,t) \nonumber \\&= \langle W^Q \rangle + \langle W^{CD} \rangle \nonumber \\&= \Delta F^\text {eq}+\langle W^{CD} \rangle . \end{aligned}$$

The first term $\langle W^Q \rangle$ is the amount of work that would be performed *if* the protocol were executed quasistatically, i.e., reversibly. This *quasistatic work* is simply the change in equilibrium free energy, as follows by direct substitution of Eq. (6) into the first line above. This contribution depends only on the initial and final potential and not on either (i) the sequence of intermediate distributions or (ii) the duration of the protocol.

The second contribution $\langle W^{CD} \rangle$ is the *counterdiabatic work*, and it is proportional to the global entropy production $\langle \Sigma \rangle$. Specifically, when the system begins and ends in equilibrium we have [39]:

13 $$\begin{aligned} T\langle \Sigma \rangle&= \langle W \rangle -\Delta F^\text {eq} \nonumber \\&= \langle W^{CD} \rangle , \end{aligned}$$

where $\langle \Sigma \rangle \ge 0$ quantifies the net change in the system’s entropy and its thermal surroundings. Previous analyses of counterdiabatic protocols recognized this additional work as the dissipated work [20].

In Eq. (12), the quasistatic work is fixed and the counterdiabatic work gives the path-dependent dissipated work:

$$\begin{aligned} \langle W^{CD} \rangle =\langle W_\text {diss}\rangle \end{aligned}$$

required to complete the transformation in finite time. Throughout, the counterdiabatic and dissipated works are treated as the same. Thus, all dependence on intermediate details is captured by $\langle W^{CD} \rangle$. This quantity is our principal focus and, as we now show, it scales particularly simply with system size and computation time.

We note that Eqs. (12) and (13), along with the inequality $\langle \Sigma \rangle \ge 0$, generalize to transformations between nonequilibrium states, with $\Delta F^\text {eq}$ replaced by the recoverable nonequilibrium free energy $\Delta F^\text {neq}$; see Refs. [2, 40, 41] for details. We use this generalized result in Sec. 4 when discussing counterdiabatic erasure.

While we derived our results within Ref. [13]’s framework, similar results were obtained in other contexts. Reference [42] argued that a flow field, like our *v*(*x*, *t*), could be designed to force a system to follow a target equilibrium distribution; see Eq. (15) therein. Reference [17], establishing a refinement of the second law of thermodynamics, also exploited a deterministic velocity field; their Eq. (2) is equivalent to our Eq. (10). And, Ref. [20] developed a counterdiabatic method they call “shortcuts to isothermality”; their Eq. (12) is equivalent to our Eq. (11).

Finally, we obtain a compact expression for the counterdiabatic work:

14 $$\begin{aligned} \langle W^{CD} \rangle&= \int _0^\tau dt \int _{-\infty }^\infty dx \Pr (X_{t}=x) \partial _t V^{CD}(x,t) \nonumber \\&= -\int _0^\tau dt \int _{-\infty }^\infty dx \, \partial _t \Pr (X_{t}=x) V^{CD}(x,t) \nonumber \\&= \mu \int _0^\tau dt \int _{-\infty }^\infty dx \, \Bigl [ \partial _x V(x,t) \Pr (X_{t}=x) + k_B T \partial _x \Pr (X_{t}=x) \Bigr ] \partial _x V^{CD} \nonumber \\&= \mu \int _0^\tau dt \int _{-\infty }^\infty dx \, \Pr (X_{t}=x) \left[ \partial _x V^{CD} \right] ^2 \nonumber \\&= \mu ^{-1} \int _0^\tau dt \, \left\langle \left[ v(x,t) \right] ^2 \right\rangle . \end{aligned}$$

Here, we integrated by parts in time to get to the second line and in space to get to the third line. Then, we used Eqs. (6), (9), and (10) to complete the calculation.

Equation (14) is a slight permutation of the expression for entropy production found in Ref. [43]. It is also equivalent to Eq. (2.20) of Ref. [17], where this result was used to obtain the minimally dissipative protocol for transforming from a given initial distribution $\Pr (X_0)$ to a given final distribution $\Pr (X_\tau )$, at fixed $\tau$. Namely, the minimally dissipative protocol is one for which the trajectories generated by the flow field *v*(*x*, *t*) evolve linearly with time. The velocity of level curves of the cumulative distribution function stay constant, meaning that thermodynamically optimal control yields linear interpolation from the initial *C*(*x*, 0) to final $C(x,\tau )$; see Refs. [17, 44] for further details.

### Time Reversal Symmetries

Decomposing potential energy and work into quasistatic and counterdiabatic components leads to terms with different time-reversal symmetries. To reverse a computation—creating, for example, a bit rather than erasing one—we choose the reverse trajectory distribution $\Pr (X^\text {reverse}_t=x)=\Pr (X^d_{\tau -t}=x)$. Substituting this into the quasistatic potential in Eq. (6) leads to reverse temporal ordering:

15 $$\begin{aligned} V^Q_\text {reverse}(x,t)=V^Q(x,\tau -t) . \end{aligned}$$

However, substituting into the counterdiabatic potential of Eq. (11) and setting $t'=\tau -t$ in the integration leads to a flipped sign:

16 $$\begin{aligned} V^{CD}_\text {reverse}(x,t)=-V^{CD}(x,\tau -t) . \end{aligned}$$

Then, putting each of these into Eq. (12)’s expression for work production shows that the change in free energy is inverted:

17 $$\begin{aligned} \Delta F^\text {eq}_\text {reverse}=-\Delta F^\text {eq} , \end{aligned}$$

as expected. The counterdiabatic dissipated work is the same, though, since signs cancel:

18 $$\begin{aligned} \langle W^{CD}_\text {reverse} \rangle = \langle W^{CD}\rangle . \end{aligned}$$

Thus, while the Landauer contribution to the work investment changes sign under reversed computation, since state-space contraction becomes expansion, the actual dissipation—unrecoverable component of work investment—remains the same for finite-time operations.

### System-Size and Computation-Rate Dependence

A protocol’s *duration* $\tau$ is the time over which the Hamiltonian varies. For our one-dimensional system, we define a characteristic *system length* *L* reflecting the extent of the desired probability distribution’s support. Since we wish to capture only the distribution’s bulk and not the support’s absolute extent, there are many ways to define this length. A candidate is the initial variance:

$$\begin{aligned} L= \sqrt{\int _{-\infty }^{\infty }dx \Pr (X^d_0{=}x)x^2 - \left( \int _{-\infty }^{\infty }dx \Pr (X^d_0{=}x)x \right) ^2} . \end{aligned}$$

The particular form is somewhat arbitrary. All we ask is that *L* scale appropriately when transforming the distribution. With these definitions in hand, we can analyze how the protocol and dissipation change under rescalings.

Consider the probability trajectory $\{\Pr (X^d_t=x): ~t \in (0,\tau )\}$ and a system of length *L*, yielding the control protocol $V(x,t)=V^Q(x,t)+V^{CD}(x,t)$. To preserve the probability trajectory shape while changing the duration to $\tau '$ and length to $L'$, we introduce a new desired trajectory:

$$\begin{aligned} \Pr (X_t^{d \prime }{=}x)=\Pr (X^d_{\tau t/\tau '}{=}Lx/L')\frac{L}{L'} . \end{aligned}$$

This stretches the original distribution’s support by a factor $L'/L$ and increases the computation rate by a factor $\tau /\tau '$.

In the expression for the resulting counterdiabatic control protocol:

$$\begin{aligned} V'(x,t)=V'^Q(x,t)+V'^{CD}(x,t) , \end{aligned}$$

we define a new quasistatic potential as the similarly-scaled version of the original:

$$\begin{aligned} V^{Q\prime }(x,t)=V^Q(Lx/L',\tau t / \tau ') . \end{aligned}$$

The associated equilibrium free energy is expressed in terms of the original free energy:

19 $$\begin{aligned} F^{\text {eq}\prime }(t)&= -k_\text {B}T \ln Z'(t) \nonumber \\&= -k_\text {B}T \ln \int _{-\infty }^{\infty } dx e^{-V^{Q\prime }(x,t)/k_\text {B}T} \nonumber \\&= -k_\text {B}T \ln \int _{-\infty }^{\infty } dx' \frac{L'}{L}e^{-V^{Q}(x',\tau t/\tau ')/k_\text {B}T} \nonumber \\&= k_\text {B}T \ln \frac{L}{L'} +F^\text {eq}(\tau t/\tau ') , \end{aligned}$$

where the third line comes from substituting $x = x' L'/L$. Equation (19) implies:

$$\begin{aligned} \Delta F^{\text {eq} \prime } = F^{\text {eq} \prime }(\tau ')-F^{\text {eq} \prime }(0) = \Delta F^\text {eq} . \end{aligned}$$

Hence, the quasistatic work is the same for protocols with different durations and lengths:

$$\begin{aligned} \langle W^{Q \prime } \rangle =\langle W^{Q} \rangle . \end{aligned}$$

The counterdiabatic contributions, however, yield meaningful differences when changing system length or protocol duration. Substituting the rescaled probability trajectory into the expression for counterdiabatic potential in Eq. (11), we find:

$$\begin{aligned} V^{CD \prime }(x,t)&= \frac{1}{\mu } \int _{0}^x \int _{-\infty }^{x'} \frac{\partial _t\Pr (X^{d \prime }_t=x'')}{\Pr (X^{d\prime }_t=x')} dx'' dx' \\&= \frac{1}{\mu } \int _{0}^x \int _{-\infty }^{x'} \frac{\partial _t \Pr (X^{d}_{\tau t/ \tau '}=Lx''/L')}{\Pr (X^{d}_{\tau t/ \tau '}=Lx'/L')} dx'' dx' \\&= \frac{1}{\mu } \frac{L^{\prime 2}}{L^2} \int _{0}^{Lx/L'} \int _{-\infty }^{x'''} \frac{\partial _{t'}\Pr (X^{d}_{t'}=x'''') \partial _t t'}{\Pr (X^{d}_{t'}=x''')} dx'''' dx''' \\&= \frac{\tau L^{\prime 2}}{ \tau ' L^2}V^{CD}(Lx/L',\tau t/ \tau ') , \end{aligned}$$

using the substitutions $t' = \tau t /\tau '$, $x'''=Lx'/L'$, and $x''''=Lx''/L'$. Thus, the counterdiabatic potential scales as the square of the length of the information storage device and as the inverse of the protocol duration. Equivalently, the additional nonequilibrium force $F^{CD}(x,t)=-\partial _x V^{CD}(x,t)$ applied to the system scales as the computation rate and square of the system size.

For the counterdiabatic work we similarly find:

$$\begin{aligned} \langle W^{CD \prime } \rangle&= \int _{0}^{\tau '} \!\!\!\! dt \int _{-\infty }^\infty \!\!\!\! dx \Pr (X^{d\prime }_{t}=x) \partial _t V^{CD \prime }(x,t) \\&= \frac{\tau L^{\prime 2}}{ \tau ' L^2} \int _{0}^{\tau '} \!\!\!\! dt \int _{-\infty }^\infty \!\!\!\! dx \frac{L}{L'} \Pr \left( X^{d}_{\tfrac{\tau }{\tau '} t } \!\! = \!\! \frac{L}{L'}x \right) \partial _t V^{CD}\left( \frac{L}{L'}x, \frac{\tau }{\tau '} t \right) \\&= \frac{\tau L^{\prime 2}}{ \tau ' L^2} \int _{0}^{\tau } \frac{\tau '}{\tau }dt' \int _{-\infty }^\infty \frac{L'}{L}dx' \frac{L}{L'}\Pr (X^{d}_{t'}=x') \partial _t V^{CD}(x',t') \\&= \frac{\tau L^{\prime 2}}{ \tau ' L^2} \frac{\tau '}{\tau } (\partial _t t')\int _{0}^{\tau } \!\!\!\! dt' \int _{-\infty }^\infty \!\!\!\! dx' \Pr (X^{d}_{t'}=x') \partial _{t'} V^{CD}(x',t') \\&= \frac{\tau L^{\prime 2}}{ \tau ' L^2} \langle W^{CD} \rangle . \end{aligned}$$

And so, too, the dissipated counterdiabatic work scales as system length squared and linearly with computation rate.

Dissipation has been shown to scale with driving rate in far-from-equilibrium operations [15, 17–21] and the counterdiabatic potential was shown to scale similarly [20]. The length-squared scaling has also been observed in the linear-response regime, when systems are close to equilibrium [11]. That said, the simultaneous scaling far-from-equilibrium of both the dissipation and counterdiabatic potential are novel and appear here in a unified framework. This work, in turn, is proportional to the entropy production, so we find that the entropy production obeys a similar scaling:

20 $$\begin{aligned} \langle \Sigma ' \rangle&= \frac{\langle W^{CD \prime } \rangle }{T} \nonumber \\&= \frac{\tau L^{\prime 2}}{ \tau ' L^2}\langle \Sigma \rangle . \end{aligned}$$

### Efficient Protocols

When changing the protocol duration $\tau \rightarrow \tau '$ and system length $L \rightarrow L'$ of a desired distribution trajectory $\{\Pr (X^d_t)\}$, the counterdiabatic control becomes:

$$\begin{aligned} V'(x,t) = V^Q \left( \frac{L}{L'}x,\frac{\tau }{\tau '}t \right) + \frac{\tau L^{\prime 2}}{ \tau ' L^2} V^{CD} \left( \frac{L}{L'}x,\frac{\tau }{\tau '}t \right) , \end{aligned}$$

where $V^Q(x,t)$ and $V^{CD}(x,t)$ are the original quasistatic and counterdiabatic potential energies. This leads to the work investment:

$$\begin{aligned} \langle W'\rangle =\Delta F^\text {eq}+\frac{\tau L^{\prime 2}}{ \tau ' L^2} \langle W^{CD} \rangle , \end{aligned}$$

where $\Delta F^\text {eq}$ is the original change in free energy and $\langle W^{CD} \rangle$ is the original nonequilibrium addition to work.

The above scaling relation is suggestive, and it is worth considering how it applies to maximally efficient computations. While the following does not recount the steps described by Aurell et al. to determine these minimally dissipative protocols [16, 17], one can nevertheless see how such protocols change as time and length scales change. We use a simple counterfactual argument, described below, to show that the minimum cost of computing scales as $L^2/ \tau$.

The first step in addressing minimum dissipation protocols is to recognize that counterdiabatic protocols in ${\mathcal {X}}={\mathbb {R}}$ are uniquely determined by the distribution trajectory. Since a computation maps an initial equilibrium distribution $\Pr (X_0)$ to a final one $\Pr (X_\tau )$, there are many compatible distribution trajectories that evolve continuously from the initial to the final distribution. A minimally-dissipative distribution trajectory $\Pr (X_{t,\text {min}})$ has a corresponding $V_\text {min}(x,t)=V^Q_\text {min}(x,t)+V^{CD}_\text {min}(x,t)$ that yields the minimum work:

$$\begin{aligned} \langle W^{CD}\rangle _\text {min} = \min \{\langle W^{CD}\rangle : \Pr (X_{0,\tau }^d)=\Pr (X_{0,\tau }) \} . \end{aligned}$$

Since quasistatic work is identical for all such protocols, up to an instantly recoverable additional energy, this condition also minimizes invested work.

Changing protocol duration $\tau \rightarrow \tau '$ and initial and final system length—viz., $\Pr (X'_0=x)=\Pr (X_0=Lx/L')$ and $\Pr (X'_{\tau '}=x)=\Pr (X_\tau =Lx/L')$—we can determine how the minimally-dissipative distribution trajectory changes, as well the minimum dissipation. A natural guess for the minimally-dissipative trajectory is to take the scaled minimal distribution:

$$\begin{aligned} \Pr (X^{\prime }_{t}=x)=\Pr (X_{\tau t/\tau ',\text {min}}=Lx/L')\frac{L}{L'} , \end{aligned}$$

which satisfies:

$$\begin{aligned} \big \langle W^{CD \prime } \big \rangle = \frac{\tau L^{\prime 2}}{ \tau ' L^2} \big \langle W^{CD} \big \rangle _\text {min} . \end{aligned}$$

(See Sect. 3.4.)

If this proposed trajectory is not minimally dissipative, then there is another trajectory $\{\Pr (X^\prime _{t,\text {min}})\}$ that dissipates work $\langle W^{CD \prime } \rangle _\text {min} < \langle W^{CD \prime } \rangle$. However, if this were the case, then we could reverse the duration and size scalings $\tau ' \rightarrow \tau$ and $L' \rightarrow L$ on that trajectory to generate the dissipation:

$$\begin{aligned} \frac{\tau ' L^2 }{\tau L^{\prime 2}} \big \langle W^{CD \prime } \big \rangle _\text {min}&< \frac{\tau ' L^2 }{\tau L^{\prime 2}} \big \langle W^{CD \prime } \big \rangle \\&= \frac{\tau ' L^2 }{\tau L^{\prime 2}} \frac{\tau L^{\prime 2}}{ \tau ' L^2} \big \langle W^{CD} \big \rangle _\text {min} \\&=\big \langle W^{CD} \big \rangle _\text {min} . \end{aligned}$$

This is a contradiction, since it states that it is possible to dissipate less than the minimal dissipation for the original computation that evolves the distribution between $\Pr (X_0)$ and $\Pr (X_\tau )$. We conclude that the spatially- and temporally-scaled minimally dissipative distribution trajectories are themselves minimally dissipative.

This agrees nicely with the minimally-dissipative mass transport described by Ref. [17], in which probability mass takes a linear path between initial and final positions. Paralleling other approaches in the restricted near-equilibrium regime [7, 45], we showed that optimal control discussed above and derived in Ref. [17], if found in one setting, can be scaled to express optimal control given other constraints on space and time. Moreover, it gives the temporal scaling of the minimally-dissipative control protocol:

21 $$\begin{aligned} \begin{aligned} V^\prime _\text {min}(x,t) = V^Q_\text {min}(Lx/L',\tau t/\tau ') + \frac{\tau L^{\prime 2}}{ \tau ' L^2} V^{CD}_\text {min}(Lx/L',\tau t/\tau ') , \end{aligned} \end{aligned}$$

and of the minimum work production:

22 $$\begin{aligned} \langle W^\prime \rangle _\text {min} = \Delta F^\text {eq} +\frac{\tau L^{\prime 2}}{ \tau ' L^2}\langle W^{CD}\rangle _\text {min} . \end{aligned}$$

The second term in Eq. 22 is the dissipated (counterdiabatic) work and, once again, agrees with Ref. [17]’s optimal transport results, that derived an inverse relationship between the dissipation and the time scale of computation.

Equation 22 matches independent analyses on the scaling of dissipated work for optimal control [7]. However, the present results apply more generally: without restricting control parameters—all potential landscapes are allowed—and, crucially, beyond linear response. This perhaps explains the puzzle that the results derived assuming linear response [6, 7] appeared to work outside of those constraints.

Additional, key differences should be highlighted. First, under geometric control the same control path (through the space of potential landscapes) is followed regardless of $\tau$. As a result, the minimally-dissipative control simply scales as [6]:

23 $$\begin{aligned} V'_\text {min}(x,t)=V_\text {min}(x, \tau t/ \tau ') . \end{aligned}$$

By contrast, Eq. (21) reveals a different scaling for the minimally-dissipative counterdiabatic protocol, in which the magnitude of the counterdiabatic potential $V_\mathrm{min}^{CD}$ is enhanced by a factor $\tau /\tau ^\prime$, relative to the quasistatic potential $V_\mathrm{min}^Q$.

Moreover and constructively, counterdiabatic protocols allow a control engineer to specify exact initial and final conditions. This flexibility is key to, for instance, matching gate outputs to gate inputs when composing logic circuits. In other treatments, such as the geometric control setting, initial and final conditions cannot be set arbitrarily, but must be inferred from dynamics—a rather awkward requirement for design.

In short, counterdiabatic control of Fokker–Planck dynamics in one dimension gives precise control over distributions and yields constructive methods for designing control protocols. The resulting energetic costs depend simply on overall system temporal and spatial scales, revealing a tradeoff beyond that between a computation’s information processing and energy cost.

## Counterdiabatic Erasure

We now apply the counterdiabatic approach to the paradigmatic example of erasing a bit of information in a metastable system. The analysis exposes new elements in the resource tradeoffs that arise in thermodynamic computing.

### Nonequilibrium Information Storage

Quickly shifting probability distributions in one-dimensional nonlinear Langevin systems gives a physical implementation of fast logical operations. For instance, erasure is an essential part of most computations and can be achieved by controlling a double-well potential landscape [46, 47]. Landauer stated that erasure requires dissipating at least $k_\text {B}T \ln 2$ of work—a cost deriving from the microstate space contraction induced by the logically irreversible operation [1]. This bound is indeed achievable in the present setting, but only in the limit of quasistatic operations, where zero entropy is produced globally. That is, it is achievable only in infinite time. For finite-time processes, there is dissipation and, thus, additional work required for erasing a bit of information [7, 46, 47].

We will now derive the same additional cost for finite-time erasure, departing from prior treatments within the framework of geometric control in linear response [6–8]. The latter finds thermodynamically efficient paths between different control parameters; in our case given by the potential energy landscape *V*(*x*, *t*). In these cases, it is assumed that systems are near-enough to equilibrium that the distribution—in our case $\Pr (X_t)$—is nearly determined by the Boltzmann distribution. In this, erasure fidelity is approximately inferred rather than designed into the system. Instead, paralleling optimal transport under Fokker–Planck dynamics [16, 17, 38, 44], we start by determining the initial and final distributions and, in this way, exactly specify the fidelity of erasure, instead of merely recreating it. This strategy allows one to depart arbitrarily far from equilibrium in the path between initial and final states.

This section provides a detailed analysis of thermodynamic resources for a desired accuracy level of information processing. However, unlike the strategy of optimal transport [16, 17, 38, 44], that allows for any distribution trajectory, we limit our consideration to *metastable distributions*. As described shortly, these distributions use the system’s natural information storing capacity. While this restriction leads to dissipation beyond that achievable through optimal transport, it leads to a decomposition of the dissipation into functionally relevant terms. Within this class of computations, it is possible to design a protocol that gives perfect erasure in finite time and at finite cost. This demonstrates that, while alternate computational frameworks have a divergent error-dissipation tradeoff [28, 48], counterdiabatic computing allows for zero-error logical operations without divergent energy costs.

The expression for the counterdiabatic potential Eq. (11) specifies how to design a protocol *V*(*x*, *t*) that maintains the distribution $\Pr (X_t)$ exactly in a desired distribution $\Pr (X^d_t)$ over the interval $t \in (0,\tau )$. However, we must also consider how to use the map to informational states $c:{\mathcal {X}} \rightarrow {\mathcal {Y}}$ to perform useful and robust computation.

One strategy is to design the energy landscape such that physical states $x \in {\mathcal {X}}$ in one informational state $y \in {\mathcal {Y}}$ rarely transition to different informational states $y' \ne y$. This allows the information processing device to remain in a passive “default” state while retaining the information of its computation for long times, regardless of the outcome.

In contrast, if a computation is designed such that the equilibrium distribution $\Pr (X^\text {eq}_t)$ exactly matches the desired distribution $\Pr (X^d_t)$ after the computation, with $t>\tau$, then the energy landscape is given by $V(x,t)=F^\text {eq}(t)-k_\text {B}T \ln \Pr (X^d_t)$ for $t \ge \tau$. Hamiltonian control of the system is the external driving of the system, determined in experimental systems perhaps by a preprogrammed virtual potential [47] or time varying magnetic fluxes applied to the system [48]. Thus, the potential energy landscape can be thought of as the external configuration of our memory storage device, which the experimenter can control directly.

**Fig. 2:**
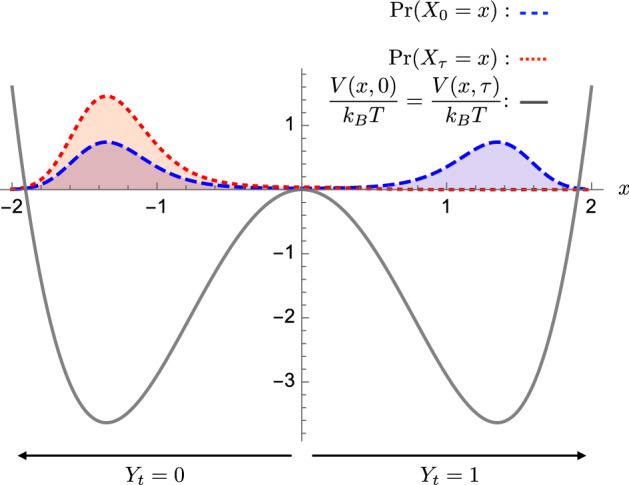
Default energy landscape: A double-well potential that stores many different distributions $\Pr (Y_t)$ over the informational states $Y_t \in \{0,1\}$. For protocols that process the information in the distributions over the times $t \in (0,\tau )$, the energy landscape is set to be the same at the beginning and end, shown by the gray curve $V(x,0)=V(x,\tau )$. The equilibrium distribution, delineated by the dashed blue curve, gives equal probabilities for the informational states: $\Pr (Y_0=0)=\Pr (Y_0=1)=1/2$. This is the initial distribution for the system $\Pr (X_0)$ in this case. It stores ${\text {H}}[\Pr (Y_0)] = 1$ bit of information, where ${\text {H}}[Z]$ is the Shannon information of random variable *Z* [49]. The red curve $\Pr (X_\tau =x)$ gives unit probability of informational state 0 ($\Pr (Y_\tau =0)=1$) and is the distribution of the system after an effective erasure protocol. Its Shannon information vanishes and so the initial and final distributions represent bit erasure. The final distribution $\Pr (X_\tau )$ is out of equilibrium, but the energy barrier between the two informational states keeps it nearly fixed for short times. This distribution, as well as many other nonequilibrium distributions, are metastable and will slowly relax to equilibrium

If the distribution is allowed to relax to equilibrium, the relevant information about the computation is stored in the memory device’s external configuration *V*(*x*, *t*) (our control). This means that it is unnecessary to track the actual physical distribution $\Pr (X_t)$. By choosing a default energy landscape for which metastable physical distributions persist in time, the computational device can robustly store information as shown in Fig. 2. This avoids explicitly encoding the computation’s outcome distribution in the energy landscape and thus the external configuration. In this way, information in an initial distribution can be preserved through a sequence of metastable computations and ultimately influence the output.

To experimentally test Landauer’s prediction [1], Ref. [47] employed a protocol that starts and ends in a symmetric double-well potential, where each well is interpreted as a distinct mesoscopic informational state: $Y_t = 0$ or $Y_t = 1$. Such a potential stores informational states determined by the probability $\Pr (Y_t=0)$ to realize the informational state 0. Following this setup, if we start and end in a symmetric bistable potential:

24 $$\begin{aligned} V(x,0)&= V(x,\tau ) \nonumber \\&=\alpha x^4- \kappa x^2 , \end{aligned}$$

then, at a temperature *T*, the equilibrium distribution:

25 $$\begin{aligned} p(x)&\equiv \Pr (X_{\{0,\tau \}}^\text {eq}=x) \nonumber \\&= \frac{e^{-V(x,0)/k_\text {B}T}}{Z} , \end{aligned}$$

is bimodal; see the blue dashed curve in Fig. 2. While this distribution is exactly stationary (when the potential is held fixed), we can construct other distributions that are (temporarily) effectively stationary, such as that given by the dotted red curve shown in Fig. 2. This has the same shape as the equilibrium distribution over the interval $(-\infty ,0)$, but vanishes outside. By specifying a time-dependent *bit bias* probability $\Pr (Y_t=0)=b(t)$, we fully specify a metastable physical distribution [50]:

26 $$\begin{aligned} \Pr (X^\text {met}_t=x) = {\left\{ \begin{array}{ll} p(x)\cdot 2b(t) &{} \text { if } x\le 0 \\ p(x)\cdot 2(1-b(t)) &{} \text { if } x> 0 \end{array}\right. }. \end{aligned}$$

We take this distribution to be our desired distribution $\Pr (X^d_t)$, which in turn defines the quasistatic potential $V^Q(x,t)$.

Figure 2 shows the metastable distributions before (blue dashed curve) and after (red dotted curve) an erasure protocol, where the initial distribution is unbiased $b(0)= 1/2$ and the final distribution is totally biased $b(\tau )=1$. The energy barrier between informational states 0 and 1 inhibits large probability flow between the two local equilibria. That is, these distributions relax to a global equilibrium very slowly, depending on barrier height relative to $k_\text {B}T$ [50]. Thus, these metastable states robustly store nonequilibrium informational states and provide a basis for information processing by a controlled double-well potential.

**Fig. 3:**
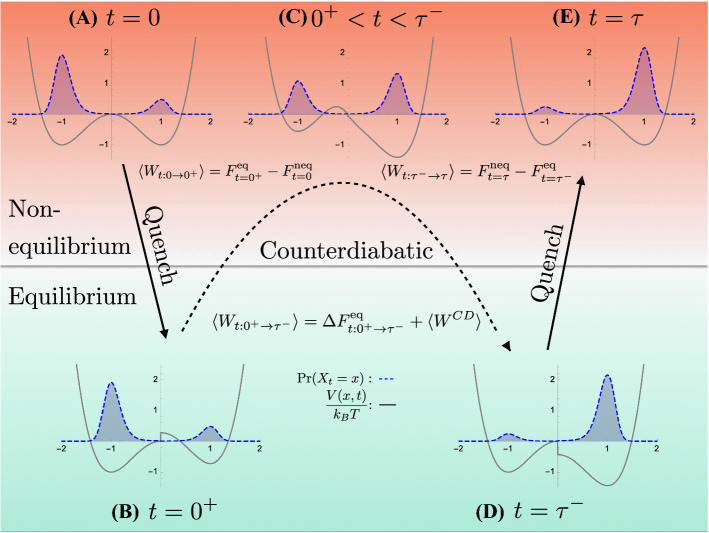
Counterdiabatic information processing in three steps: Distribution $\Pr (X_t=x)$ shown with blue dashed curves and energy landscapes *V*(*x*, *t*) shown by the gray curves. First, the information landscape is instantaneously changed to fit the starting distribution from stage (A) to stage (B). These share the same distribution but have different energy landscapes. Second, the counterdiabatic protocol is applied to take the system from the equilibrium distribution at stage (B) to that shown in stage (D), passing through nonequilibrium distributions driven from equilibrium by the counterdiabatic potential; such as that shown in stage (C). Third, the last quench step instantaneously takes the system from equilibrium stage (D) to the nonequilibrium metastable stage (E). All three transitions are labeled with the associated work investment

### Counterdiabatic Information Processing

We now consider how to use counterdiabatic driving to steer the system through a sequence of metastable states specified by a given time-dependent bit bias *b*(*t*), with the symmetric initial and final energy landscape of Eq. (24). Despite the symmetric initial and final configurations of the memory device, this modified counterdiabatic control allows for $b(0) \ne 1/2$ and $b(\tau ) \ne 1/2$.

Since the initial and final metastable states are out of equilibrium with respect to the symmetric potential $V(x,0)=V(x,\tau )=\alpha x^4-\kappa x^2$ (Fig. 2), we must modify the counterdiabatic protocol described in Sec. 3.2, as it was developed for transitions between initial and final equilibrium distributions. Two additional steps are needed, each a quench, as shown in Fig. 3. (*Quench* here means a nearly instantaneous change in the Hamiltonian [51], as opposed to a nearly instantaneous change in temperature, as often intended.) These quenches are added to make the quasistatic potential $V^Q(x,t)$ match the equilibrium distribution of the desired metastable distribution $\Pr (X^\text {met}_t)$ over the open time interval $t \in (0 ,\tau )$.

Specifically, for $t \in (0,\tau )$ we set:

$$\begin{aligned} V^Q(x,t)=F^\text {eq}(t)-k_\text {B}T \ln \Pr (X^\text {met}_t=x). \end{aligned}$$

Hence, at $t=0$ the energy landscape undergoes a quench from the symmetric potential *V*(*x*, 0) to the asymmetric potential $V^Q(x,0)$. We then add the counterdiabatic term:

$$\begin{aligned} V^{CD}(x,t) =\frac{1}{\mu } \int _{0}^x \int _{-\infty }^{x'} \frac{\partial _t\Pr (X^\text {met}_t=x'')}{\Pr (X^\text {met}_t=x')}dx'' dx', \end{aligned}$$

such that the overall potential becomes:

$$\begin{aligned} V(x,t)=V^{Q}(x,t)+V^{CD}(x,t) . \end{aligned}$$

For $t\in (0,\tau )$ the system evolves through the desired sequence $\Pr (X^\text {met}_t=x)$, corresponding to the equilibrium states of $V^Q(x,t)$. At the end of the protocol the system undergoes another quench, from the asymmetric potential $V^Q(x,\tau ^-)$ to the symmetric potential $V(x,\tau )$. In this way, we drive the system through a sequence of metastable distributions with precise control of the bit bias *b*(*t*).

Although the protocol just described pertains to the specific case of a double well, the procedure of quenching, controlling counterdiabatically, and then quenching again is a general technique for evolving between nonequilibrium distributions in finite time. For such a computation, the total work simplifies to the net change in nonequilibrium free energy plus the counterdiabatic work:

27 $$\begin{aligned} \langle W \rangle = \Delta F^\text {neq}+\langle W^{CD}\rangle , \end{aligned}$$

as shown in Fig. 3. The change in nonequilibrium free energy is given by the sum of the quasistatic work and the quenching work [2, 40]. For the metastable distributions we discussed, where each informational state contributes the same local free energy, $\Delta F^\text {neq}$ reduces to the change in the Shannon entropy of the information variable [2]:

28 $$\begin{aligned} \Delta F^\text {neq}&= k_\text {B}T \ln 2 \left( {\text {H}}[Y_0]-{\text {H}}[Y_\tau ] \right) . \end{aligned}$$

Since $\langle W^{CD}\rangle = T \langle \Sigma \rangle \ge 0$ (see Sect. 3.2), Eqs. (27) and (28) produce the generalized form of Landauer’s bound [2, 52, 53]:

29 $$\begin{aligned} \langle W\rangle \ge k_\text {B}T \ln 2 \left( {\text {H}}[Y_0]-{\text {H}}[Y_\tau ] \right) , \end{aligned}$$

which takes on the familiar form, $\langle W \rangle \ge k_\text {B}T \ln 2$, when starting with fully randomized bits, $b(0)=1/2$ and when the operation’s fidelity is perfect, $b(\tau )=1$. As we shall see, even if the Landauer bound cannot be achieved in finite time, perfect fidelity can be achieved in finite time with finite work.

The amount of entropy produced—$\langle \Sigma \rangle = \langle W^{CD} \rangle /T$—reflects the additional cost beyond Landauer’s bound to implement a computation in finite time. For metastable erasure in a symmetric double well, this additional cost depends on duration, system length scale, bit bias difference, and information lifetime—a measure of information storage robustness. We have already seen (Sect. 3.4) that the value of $\langle \Sigma \rangle$ scales as the inverse of the protocol duration $\tau$ and the square of the system characteristic length scale *L*. We now show how bit bias difference and information lifetime lead to additional energy costs.

Metastability simplifies the expression for the counterdiabatic potential, leading to simple relationships between the work, bit bias difference, and robustness of information storage. As shown in Appendix B, the counterdiabatic potential can be expressed as a product of a piecewise-continuous function and a function that depends only on the equilibrium distribution:

30 $$\begin{aligned} V^{CD}(x,t)&= h(x) \times {\left\{ \begin{array}{ll} - \partial _t \ln b(t) &{} \text { if } x\le 0 \\ - \partial _t \ln (1-b(t)) &{} \text { if } x>0 \end{array}\right. } , \end{aligned}$$

where:

$$\begin{aligned} h(x) = \frac{1}{\mu }\int _0^{|x|}dx' \frac{1}{p(x')} \int _{-\infty }^{-|x'|}dx''p(x'') \end{aligned}$$

and $p(x)=\Pr (X_0^\text {eq}=x)$ is the equilibrium distribution for the symmetric, bistable potential of Eq. (25). This result allows us to design protocols for evolving a metastable distribution from an initial bit bias $b(0)=b_i$ to any final bit bias $b(\tau )=b_f$, given a bistable potential. For instance, the choices $b_i=1/2$ and $b_f=1$ correspond to perfect erasure, where the system ends entirely in the left well.

### Tradeoffs in Metastable Symmetric Erasure

As discussed above, the equilibrium distribution *p*(*x*) and bit bias *b*(*t*) determine both the desired metastable distribution trajectory of Eq. (26) and the counterdiabatic potential of Eq. (30) that generates this evolution. Appendix B shows that the functions *p*(*x*) and *b*(*t*) are multiplicatively separable in the expression for counterdiabatic work. Specifically:

$$\begin{aligned} \langle W^{CD} \rangle =f_1[p(\cdot )]f_2[b(\cdot )] , \end{aligned}$$

where:

31 $$\begin{aligned} f_1[p(\cdot )]&= \frac{2}{\mu } \int _0^\infty dx \,p(x) \int _0^{x}dx' \frac{1}{p(x')} \int _{-\infty }^{-x'}dx''p(x'') \nonumber \\ f_2[b(\cdot )]&= \int _{0}^\tau dt \frac{ (\partial _t b(t))^2}{b(t)-b(t)^2}. \end{aligned}$$

This separability follows from the metastability and symmetry of the potential energy landscape. Notably, it leads to additional tradeoffs between dissipation, bit bias difference, and information lifetime. These go beyond the thermodynamic costs of computation rate and spatial scale.

Functional $f_1$ depends on the equilibrium distribution *p*(*x*) that, in turn, is determined by the bistable potential *V*(*x*, 0). The shape of this potential (e.g., the height of the barrier relative to the left and right minima) determines the expected “lifetime” of a stored bit, in the absence of external driving. Thus, $f_1$ contains all the dependence of the counterdiabatic work on information storage robustness.

Functional $f_2$ depends on the bit bias trajectory *b*(*t*). One can now entertain a variety of bias trajectories, using this functional to determine how the counterdiabatic work changes. However, note that one must restrict to paths for which the initial and final time-derivative vanishes $\partial _t b(t)|_{t \in \{0, \tau \}}=0$, since $\partial _t \Pr (X_t=x)_{t \in \{0, \tau \}}$ must vanish for the counterdiabatic potential itself to be zero initially and finally.

Note too that $f_1$ and $f_2$ contain the system length and protocol duration dependence, respectively. If we rescale the system spatially and the protocol temporally, we obtain the new equilibrium distribution and bias trajectory:

$$\begin{aligned} p'(x)&= \frac{L}{L'}p(Lx/L') ~\text {and}\\ b'(t)&= b(\tau t/\tau '). \end{aligned}$$

Plugging these in, we find the new functionals:

$$\begin{aligned} f_{1}[p'(\cdot )]&= \frac{L^{\prime 2}}{L^2}f_{1}[p(\cdot )] ~\text {and}\\ f_{2}[b'(\cdot )]&= \frac{\tau }{\tau '}f_{2}[b(\cdot )]. \end{aligned}$$

To further separate dependencies, we introduce unitless functionals of both bias and the default equilibrium distribution:

$$\begin{aligned} F_1[p(\cdot )]&= f_1[p(\cdot )]/L^2 ~\text {and}\\ F_2[b(\cdot )]&= f_2[b(\cdot )]\tau . \end{aligned}$$

$F_2$ captures the difference between initial and final bias without dependence on computation rate. $F_1$ captures the depth between left and right wells without dependence on the spatial scale, which also affects how long a bistable system can robustly store information.

In short, the counterdiabatic work is expressed as the product of four factors:

$$\begin{aligned} \langle W^{CD} \rangle = \frac{L^2}{\tau }F_1[p(\cdot )]F_2[b(\cdot )]. \end{aligned}$$

Since $F_1$ and $F_2$ are independent of duration and system length, this implies a five-way tradeoff between the main dependencies of computation: dissipation, duration, length, $F_1$, and $F_2$. We next study how $F_1$ and $F_2$ depend on properties of the erasure protocol, leading to a practical consequence.

### Perfect Erasure in Finite Time with Finite Work

Let us consider control protocols for which the bit bias trajectory is given by:

32 $$\begin{aligned} b(t) = b_i \cos ^2(\pi t /2\tau ) + b_f \sin ^2(\pi t /2\tau ). \end{aligned}$$

**Fig. 4:**
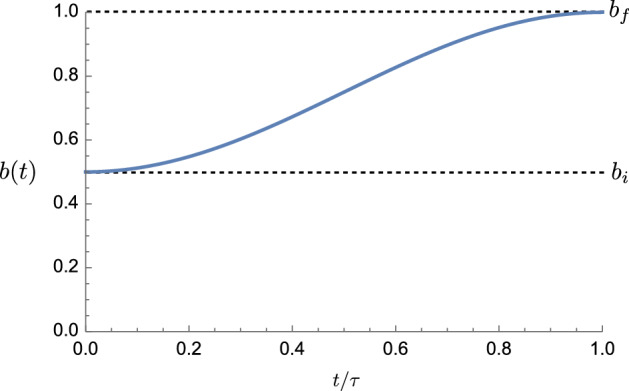
Nonlinear protocol for driving between initial bit bias $b(0)=b_i$ and final bias $b(\tau )=b_f$. The nonlinear protocol *b*(*t*) (blue curve) has zero slope initially and finally such that the counterdiabatic potential vanishes at the protocol’s beginning and end

This schedule takes the system monotonically from $b(0)=b_i$ to $b(\tau )=b_f$, as shown in Fig. 4. Since $\partial _t b=0$ at $t=0$ and $t=\tau$, the counterdiabatic potential vanishes at the initial and final times, except in the special cases that $b_i$ or $b_f$ are either 0 or 1.

As shown in Eq. (30), the multiplicative contribution of the bias trajectory to the counterdiabatic potential is $-\partial _t \ln b(t)= -\partial _t b(t) / b(t)$, if $x \le 0$, and $-\partial _t \ln (1- b(t))= \partial _t b(t) /(1-b(t))$, if $x>0$. If *b*(*t*) is 0 or 1, then one diverges. And, in the case where $\partial _t b(t)$ is zero, such as when $t=0$ or 1, using L’Hopital’s rule to evaluate the undefined ratio 0/0, leaves a term proportional to $\partial ^2_t b(t) / \partial _t b(t)$. This diverges since $\partial ^2_t b(t) \ne 0$.

The counterdiabatic potential in this case is:

33 $$\begin{aligned} \begin{aligned} V^{CD}(x,t)&= \frac{h(x)}{2 \tau } \times {\left\{ \begin{array}{ll} -\frac{(b_f-b_i)\pi \sin (\pi t/\tau )}{b_i\cos (\pi t/2\tau )^2+b_f\sin (\pi t /2\tau )^2} &{} \text { if } x\le 0 \\ \frac{(b_f-b_i)\pi \sin (\pi t/\tau )}{1-b_i\cos (\pi t/2\tau )^2-b_f\sin (\pi t /2\tau )^2} &{} \text { if } x>0 \end{array}\right. }. \end{aligned} \end{aligned}$$

**Fig. 5:**
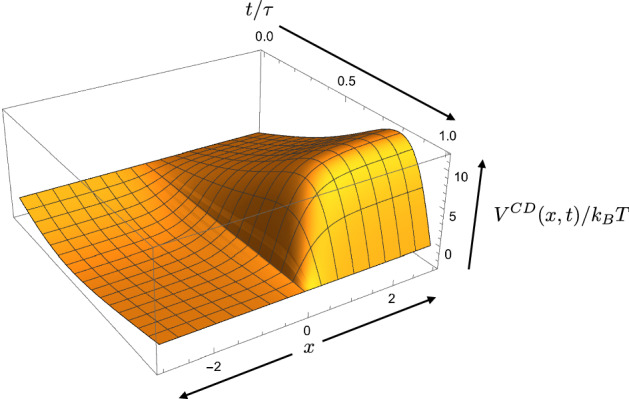
Counterdiabatic potential for the nonlinear erasure protocol of Fig. 4 that takes a bistable potential well from an initial bias $b(0) = 0.5$ to a final bias $b(\tau ) = 0.95$. For this protocol we set $\tau =1$, $\mu =1$, $k_B T=1$, $\alpha =1$, and $\kappa =2$. The counterdiabatic potential vanishes at the beginning and end, so that the system begins and ends in equilibrium

Note that the explicit dependence on duration factors out, yielding the prefactor $\tau ^{-1}$, as expected. Calculating *h*(*x*) numerically, Fig. 5 plots the counterdiabatic potential $V^{CD}(x,t)$. The nonlinear protocol begins and ends with zero counterdiabatic potential, hence the distribution begins and ends in equilibrium. This guarantees that when instantaneously changing back to the default bistable potential landscape, the work investment beyond the counterdiabatic work equals the change in nonequilibrium free energy.

Equation (30) indicates that any protocol starting or ending with all probability in a single well ($b_i = 0$, $b_i = 1$, $b_f = 0$, or $b_f = 1$) has divergent counterdiabatic potentials, since either *b*(*t*) or $1-b(t)$ vanishes. A vanishing numerator ${\dot{b}}(t)$ is no compensation, since under any number of applications of L’Hopital’s rule to evaluate convergence the numerator becomes nonzero first; it is the derivative of the denominator. We also see divergent energies in the quasistatic potential, which is proportional to $-k_B T \ln b(t)$ before the quench.

**Fig. 6:**
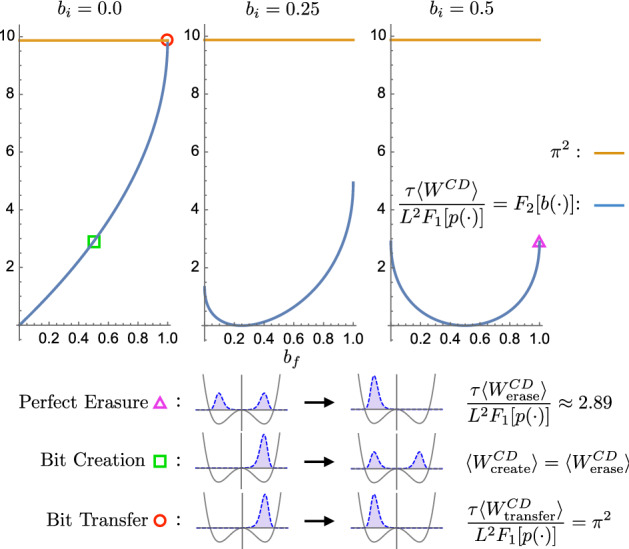
Dissipated work to execute a logical operation changes with initial bit bias $b_i$ and final bias $b_f$: Dissipated work is proportional to $F_2[b(\cdot )]$ when the duration $\tau$ and equilibrium distribution $p(\cdot )$ are held fixed. (Left) Initial bit bias $b_i=0.0$: as the probability $b_f$ of informational state 0 increases the cost of erasure increases steadily to a maximum at $b_f=1.0$. (Center) Similar behavior for an initial bias $b_i=0.25$. (Right) Fair initial distribution $b_i=0.5$. These plots highlight the finite costs for finite-time basic bit operations. The pink triangle identifies the finite dissipation of perfect erasure, changing from an initially uniform distribution over 0 and 1 to all 0. The green square identifies creating an uncertain bit from a certain bit state 0. Since it is the reverse of perfect erasure, this exhibits the same dissipated counterdiabatic work. Last, the red circle identifies the dissipation cost of transferring a bit from 1 to 0

Despite this discomforting divergent energy, the situation of perfect erasure is not inaccessible. In much the same way that quasistatic perfect erasure requires only $k_B T \ln 2$ average work production, we find through numerical calculations that a counterdiabatic potential $V^{CD}(x,t)$ that starts and ends at zero can perform perfect erasure in finite time with finite work, because the probability of high-energy states vanishes. If the thermodynamic-computing designer wishes to avoid a divergent final potential, they can approach perfectly faithful erasure asymptotically while keeping the final state in equilibrium, because $V^{CD}(x, \tau )=0$ for all $b_f \ne 0,1$. As the final bias $b_f$ approaches 1, the resulting work approaches a constant value but the system approaches perfect erasure, as shown in the rightmost plot of Fig. 6.

To study the dependence of dissipated work on initial and final bias, $b_i$ and $b_f$, we substitute the nonlinear bias function, Eq. (32), into functional $F_2[b(\cdot )]$. This functional is proportional to the dissipated work with the default distribution *p*(*x*) and duration fixed. This allows us to determine the thermodynamic cost of the basic computations on a single bit.

Figure 6 shows numerical estimates of $F_2[b(\cdot )]$ for three different initial biases $b_i=0.0$, 0.25, and 0.5. We see that the dissipation increases with the magnitude of the bias difference $|b_i-b_f|$. However, we also see that the dissipated work is bounded, since $F_2[b(\cdot )]$ is bounded by $\pi ^2$. This means that a variety of single-bit operations can be executed in finite time with finite dissipation, including perfect erasure.

Figure 6 highlights three points corresponding to *perfect erasure*, *bit creation*, and *bit transfer*. Perfect erasure, identified by the pink triangle, corresponds to starting with an unbiased state ($b_i=0.5$), then increasing the final bias to $b_f=1.0$. Since $F_2[b(\cdot )]$ converges to $\approx 2.89$, perfect erasure can be executed with finite work in finite time. Perfect bit creation, extracting a random bit ($b_f=0.5$) from a fixed bit ($b_i=0.0$), has the reverse distribution trajectory of perfect erasure. So, the dissipated counterdiabatic work is the same $\langle W^{CD}_\text {create}\rangle =\langle W^{CD}_\text {erase} \rangle$. Finally, the maximally-dissipative protocol, labeled with a red circle, corresponds to transferring a stored 1 ($b_i=0.0$) to a stored 0 ($b_i=1.0$) with perfect fidelity—perfect bit transfer. It should be noted that this transfer, while mapping 1 to 0, does not map 0 to 1. That is, it is not a swap operation.

Thus, we see that the basic and useful 1-bit operations can be implemented in finite time with these counterdiabatic protocols using finite dissipated work and at high fidelity. The general behavior of the dissipation is, for each starting bias $b_i$, that it increases with the difference between initial final bias. However, a more precise characterization in terms of a *distance* measure between probability distributions was indicated in past work, which nicely matches these results.

Note that these plots are intentionally designed in a way similar to Fig. 3 of Ref. [7] and reveal similar dependence on initial and final bias. Quantitatively, the values are proportional. Reference [7] showed that optimal control in the linear response regime requires dissipated heat proportional to the square of the Hellinger distance:

34 $$\begin{aligned} K^2(b_i,b_f)=\frac{\left( \sqrt{b_i}-\sqrt{b_f}\right) ^2+\left( \sqrt{1-b_i}-\sqrt{1-b_f}\right) ^2}{2}. \end{aligned}$$

Though our chosen bit bias trajectory is not optimal, as App. B notes, numerical integration shows that the contribution to the dissipated work can be expressed:

35 $$\begin{aligned} F_2[b(\cdot )] = \pi ^2 K^2(b_i,b_f), \end{aligned}$$

where the proportionality constant $\pi ^2$ comes from analytical results for the special case when $b_i=0.0$ and $b_f=1.0$. Thus, we see that the dissipated work is proportional to a measure of the distance between initial and final distributions for this class of control protocols.

While we do not yet have an explanation of the correspondence between our far-from-equilibrium counterdiabatic estimate and the linear-response geometric-control estimate of dissipated work in finite time, the results’ similarity is suggestive. We should point out, though, that other bias trajectories could be chosen that do not produce dissipation proportional to the square of the Hellinger distance and may be less dissipative. It may be a coincidence that our chosen bit bias trajectory yielded results similar to Ref. [7]’s linear response analysis.

### Robust Information Storage Requires Work

With a potential $V(x,0)=\alpha x^4- \kappa x^2$ that stores information in metastable distributional states, that information has a finite lifetime. In this symmetric double well, with one well corresponding to informational state 0 and the other to 1, the lifetime can be quantified in terms of the average time $\langle \tau _{0 \rightarrow 1} \rangle$ it takes for a particle to switch between these states. In the overdamped regime this *information lifetime* is given by [50, 54]:

36 $$\begin{aligned} \langle \tau _{0 \rightarrow 1} \rangle = \frac{2 \pi }{\mu \sqrt{| {\ddot{V}}(x_0,0) {\ddot{V}}(x_B,0)|}} e^{\Delta E_B/k_\text {B}T}, \end{aligned}$$

where by ${\ddot{V}}(x,0)=\partial _x^2V(x,0)$ we denote the curvature of the default potential energy landscape, $x_0=-\sqrt{\kappa /2 \alpha }$ is the location of the minimum in the metastable 0 well, $x_B=0$ is the location of the barrier maximum, and $\Delta E_B=V(x_B,0)-V(x_0,0)$ is the height of the barrier above the minimum. The latter is a useful measure of the barrier’s energy scale and, thus, how robustly the potential stores information. By explicit calculation we obtain ${\ddot{V}}(x_B,0)=-2 \kappa$, ${\ddot{V}}(x_0,0)=4 \kappa$, and:

37 $$\begin{aligned} \Delta E_B = \frac{\kappa ^2}{4 \alpha } . \end{aligned}$$

Hence, the information lifetime is:

38 $$\begin{aligned} \langle \tau _{0 \rightarrow 1} \rangle = \frac{ \pi }{\mu \kappa \sqrt{2}} e^{ \kappa ^2/4 \alpha k_\text {B}T}. \end{aligned}$$

Note that $\langle \tau _{0 \rightarrow 1} \rangle$ scales as the system length *L* squared, due to the ${\ddot{V}}$ terms in Eq. (36)’s denominator. The ${\ddot{V}}$ terms are also proportional to the scale of the energy landscape, which we characterize with $\Delta E_B$. Beyond this, the information lifetime is strongly controlled by the energy scale $\Delta E_B$ in the exponential. Thus, the information lifetime has nearly exponential dependence on this energy scale:

$$\begin{aligned} \frac{\langle \tau _{0 \rightarrow 1} \rangle }{L^2} \propto \frac{e^{\Delta E_B/k_\text {B}T}}{\Delta E_B}. \end{aligned}$$

Thus, we can capture this dependence by evaluating the information lifetime and scaling by the length. Comparing $f_1[p(\cdot )]$ to $\langle \tau _{0 \rightarrow 1} \rangle$—i.e., $F_1[p(\cdot )]=f_1[p(\cdot )]/L^2$ to $\langle \tau _{0 \rightarrow 1} \rangle /L^2$—reveals an interesting correspondence between dissipation and information lifetime, as well as identifying a term that depends on the default potential’s well depth.

**Fig. 7:**
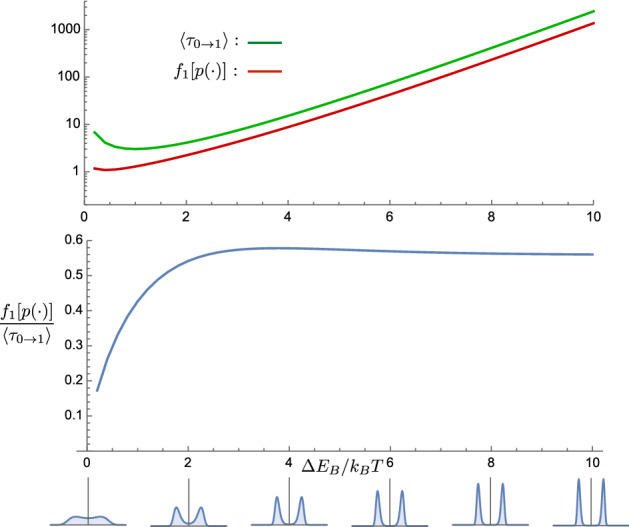
Energy barrier dependence: (Top) Changing energy barrier height $\Delta E_B$ relative to the thermal energy scale $k_\text {B}T$, $f_1[p(\cdot )]$ and so the required dissipated work increase nearly exponentially. This corresponds to an increase in the separation between the distribution in the 0 and 1 states, as shown by the six distributions along the horizontal axis (Bottom). Increasing $\Delta E_B$, greater well separation, leads to more robust information storage, as shown by the information lifetime $\langle \tau _{0 \rightarrow 1} \rangle$ (Top). (Center) Moreover, the information lifetime, which scales just below exponentially, appears to scale at the same rate as the dissipated energy when the barrier is at least twice $k_\text {B}T$. $k_\text {B}T =1$, $\alpha = \kappa /2$ (preserving the location of the potential minima), and $\mu =1$ for these calculations, while $\kappa$ is used to change the energy barrier $\Delta E_B$ as in Eq. (37)

As illustrated in Fig. 7, with increasing well depth $\Delta E_B$ the bistable distribution becomes increasingly peaked at the local minima, and the information lifetime increases nearly exponentially; as predicted by Eq. (36). Interestingly, $f_1[p( \cdot )]$, which is proportional to the excess work production required during erasure, scales at roughly the same rate as the information lifetime. Thus, the dissipated work required to erase while maintaining metastable distributions is approximately proportional to the memory’s information lifetime:The exception to this occurs for very small barrier heights, where the potential’s equilibrium distribution is not clearly bimodal and there is nearly unobstructed flow between the information states.

Figure 7 also shows that the dissipated work increases nearly exponentially with the height of the energy barrier between the wells:39The relationship between dissipation and information lifetime was studied in Ref. [55] for an erasure model in which the right side of the bistable potential is instantaneously raised—facilitating erasure to the left well—and then lowered. In contrast with our case where the required work increases nearly exponentially with energy-barrier height, in Ref. [55] the required work increases linearly with the energy barrier: $\partial \langle W \rangle /\partial \Delta E_B=1$. As a result, as one increases information storage robustness—Ref. [55]’s “reliability”—the dissipated work also scales as the barrier height. This implies a more forgiving scaling relationship between dissipation and information lifetime than the one we have derived for counterdiabatic protocols.

Unlike counterdiabatic protocols, however, Ref. [55]’s erasure protocol is not designed to precisely control the distribution and so does not allow separately identifying the dependence of dissipated work on fidelity and robustness, as done here. Thus, it is unclear how much work is required to execute perfect erasure.

Nevertheless, the relationship between dissipation and robustness found in Ref. [55] suggests that erasure efficiency may be enhanced by expanding beyond trajectory distributions that are metastable at every time step. Such distributions require a persistent energy barrier throughout the protocol. Explorations of counterdiabatic erasure protocols that eliminate the local stability of certain memory elements in a metastable system by lowering energy barriers, as done in experimental implementations of efficient Landauer erasure [47], yield less costly erasure. This has been shown explicitly in Ref. [56], which considers “bit erasure under full control”, and therefore without any constraint on intermediate probability distributions. The results show that, rather than increasing with information lifetime, counterdiabatic work and entropy production asymptote to a constant value as reliability increases.

However, full control is often inaccessible. For the metastable finite-time erasure shown here, there is a clear energy cost to robust information storage–one proportional to the information lifetime and multiplicatively separable from both the bias difference, as well as protocol duration $\tau$. Though we described how the excess work scales as the square of the length scale *L*, this dependency is directly contained in the functional $f_1[p(\cdot )]$. This reinforces the relationship to the information lifetime $\langle \tau _{0 \rightarrow 1} \rangle$, which also scales as the inverse length scale, due to the term $\sqrt{|{\ddot{V}}(x_0,0){\ddot{V}}(x_B,0)|}$. However, we also see the direct effect of the energy-barrier height $\Delta E_B$ through $F_1[p(\cdot )]$ and its near proportionality to $\langle \tau _{0 \rightarrow 1} \rangle / L^2$.

## Conclusion

Counterdiabatic control is a new tool for thermodynamic computing that executes precisely-controlled information processing in finite time at finite cost with high fidelity. It is implemented via an additional term in the potential energy—the counterdiabatic potential—that guides the microstate distribution along a path that results in the desired computation. We analyzed the work required for counterdiabatic information processing, developing a full suite of resource trade-offs. Since, as we showed, counterdiabatic protocols are the unique control that guides the system distribution along a desired trajectory, these trade-offs apply broadly to any Hamiltonian control in overdamped Fokker–Planck dynamics in one dimension. Other than the expected technical complications, the overall control strategy will generalize to higher-dimensional state spaces, as in Ref. [17].

We described how to deploy counterdiabatic protocols in combination with quenching as a general strategy for finite-time metastable information processing. Since counterdiabatic control exactly specifies the system’s final distribution, it is distinct from previous treatments of finite-time information processing using geometric control, which focused on driving an external (thermodynamic) parameter to a desired value with minimal work.

We showed that the work performed during a counterdiabatic protocol separates into the change in equilibrium free energy $\Delta F^\text {eq}$ and the counterdiabatic work $\langle W^{CD} \rangle$, which is also the dissipated work and, thus, proportional to the protocol’s entropy production. We showed that $\langle W^{CD} \rangle$ scales as the inverse of the protocol duration $\tau$—reinforcing previous analyses of finite-time thermodynamic processes that showed the work required for information processing increases with computation rate [6, 7, 45]. We also showed that dissipated work scales as the square of the system length scale *L*. That is, it is more difficult to move distributions long distances in the same finite time. The time and distance dependence together imply that going twice as far at the same speed takes twice the energy, rather similar to locomotive machines traveling long distances. This is also in agreement with microscopic experiments, such as a colloid dragged through water, for which the velocity scales as the force due to linear damping, and so the work scales accordingly: $\int dx F \propto v L=L^2/\tau$.

We then showed that counterdiabatic protocols can process information by adding quenching at a protocol’s beginning and end. Quenching allows rapidly evolving between nonequilibrium metastable states, which store information. Applying the approach, we considered a symmetric double-well system and calculated the work production for various types of finite-time bit manipulation. This analysis demonstrated that, in addition to the dependence on length scale and duration, counterdiabatic work depends on erasure fidelity and information storage robustness.

Evaluating the multiplicative component $F_2[b(\cdot )]$ of the counterdiabatic work, we found that dissipation increases with the bit bias difference between the initial and final distributions. More specifically, it is proportional to the Hellinger distance for our chosen class of control protocols. Given an initial equilibrium and unbiased metastable distribution, the closer the final metastable distribution is to giving all-0 informational states—increased erasure fidelity—the more the operation costs. However, there is an upper bound on the dissipated work. Thus, it is possible to perform perfect erasure in finite time at finite cost. It is also possible to transfer a bit in finite time with finite work, as shown in Fig. 7’s leftmost plot. Perfect fidelity, though, does not mean results are held indefinitely.

The factor $f_1[p(\cdot )]$ in the expression for the counterdiabatic work depends only on the default equilibrium distribution and so it captures the dependence on information storage robustness. That is, with increased well depth—and so metastable-state robustness—the dissipated work increases nearly exponentially. Numerical calculations demonstrate that work scales at the same rate as the information lifetime, which is the Kramers estimate [50] of the average time it takes to jump between wells.

A much richer and more detailed picture of resource tradeoffs in thermodynamic computing emerges. Most concisely, the required work decomposes as follows:

$$\begin{aligned} \langle W \rangle = k_\text {B}T \ln 2 ({\text {H}}[Y_0]-{\text {H}}[Y_\tau ]) + \frac{L^2}{\tau }F_1[p(\cdot )]F_2[b(\cdot )] . \end{aligned}$$

Landauer’s Principle for thermodynamic computing, the first term on the right, is the work required to reversibly implement a change in metastably-stored information; it is equal to the change in the physical processor’s nonequilibrium free energy. Counterdiabatic protocols complement and extend this principle. They reveal, in the second term on the right, an additional cost in the form of dissipated work that increases with the computation rate $1/\tau$, length scale squared $L^2$, fidelity through $F_2[b(\cdot )] \propto K^2(b_i,b_f)$, energy scale of information storage $\Delta E_B$ through $F_1[p(\cdot )]$, and information lifetime through the product . Thus, to achieve more efficient erasure, one must make tradeoffs by decreasing either computation rate, fidelity, length (sacrificing information lifetime), or the energy barrier (also sacrificing information lifetime). The result is a rather more complete picture of finite-time, accurate thermodynamic computing.

## Appendix A: Uniqueness of Counterdiabatic Protocols

Typically, via the Perron-Frobenius operator, the equations of motion over a space ${\mathcal {X}}$ are used to evolve the distribution $\Pr (X_t)$ over states $x \in {\mathcal {X}}$ for a time interval $t \in (0,\tau )$ from an initial distribution $\Pr (X_0)$. The inverse problem, of determining the equations of motion from the evolution of states, is more challenging. For overdamped Fokker–Planck dynamics, Ref. [13] shows how to determine the counterdiabatic control protocol $V(x,t)=V^Q(x,t)+V^{CD}(x,t)$ directly from the desired evolution of $\Pr (X^d_t)$ and, hence, determine the equations of motion. The equations of motion are specified by a changing potential landscape *V*(*x*, *t*). However, while the counterdiabatic potential is a solution to the inverse problem, given distribution trajectory $\{\Pr (X^d_t)\}$, such solutions a priori need not be unique. Here, we show that the counterdiabatic protocol is the unique protocol that generates the distribution trajectory.

We start by assuming that *V*(*x*, *t*) induces the evolution of $\Pr (X_t)$ over the time interval $(0,\tau )$. This means that it solves the Fokker–Planck equation:

A1 $$\begin{aligned} \frac{\partial \Pr (X^d_t=x)}{\partial t}&= \mu \frac{\partial }{\partial x} \left( \Pr (X^d_t=x)\frac{\partial V(x,t)}{\partial x}\right) + \mu k_\text {B}T \frac{\partial ^2 \Pr (X^d_t=x)}{\partial x^2} . \end{aligned}$$

If the potential is not the unique dynamic solving this equation, then there exists potential energy landscape:

$$\begin{aligned} V'(x,t)=V(x,t)+\Delta V(x,t) , \end{aligned}$$

that also solves this equation with nonzero $\Delta V(x,t)$. That is:

$$\begin{aligned} \frac{\partial \Pr (X_t^d=x)}{\partial t}&= \mu \frac{\partial }{\partial x} \left( \Pr (X^d_t=x)\frac{\partial [ V(x,t)+\Delta V(x,t)]}{\partial x}\right) + \mu k_\text {B}T \frac{\partial ^2 \Pr (X^d_t=x)}{\partial x^2} . \end{aligned}$$

Subtracting Eq. (A1) gives:

$$\begin{aligned} 0 = \mu \frac{\partial }{\partial x} \left( \Pr (X^d_t=x)\frac{\partial \Delta V(x,t)}{\partial x}\right) . \end{aligned}$$

Solving for the difference between the two possible solutions leads to the conclusion that all possible solutions for the difference have the form:

$$\begin{aligned} \Delta V(x,t)=C(t)+K(t) \int _0^x \frac{dx'}{\Pr (X^d_t=x')}, \end{aligned}$$

where *C*(*t*) and *K*(*t*) can vary with time, but are constant in the positional variable *x*. *C*(*t*) is an expected and trivial additional component: one can add an additional flat potential to any protocol without physical consequence beyond the change in total potential between the start and end: $C(\tau )-C(0)$. However, *K*(*t*) corresponds to an additional force—one that can have meaningful effect on the work invested during a control protocol. Thus, it appears that there are multiple ways to solve for potential energy underlying the state dynamics. However, if the state space ${\mathcal {X}}$ is truly an unbounded spatial degree of freedom, topologically equivalent to the real numbers ${\mathbb {R}}$, then the additional solutions corresponding to nonzero *K*(*t*) are unphysical.

Framed another way, these additional components in possible alternative solutions correspond to additions to the force field $F'(x,t)=F(x,t)+\Delta F(x,t)$, where:

$$\begin{aligned} \Delta F(x,t)&=-\frac{\partial \Delta V(x,t)}{\partial x} \\&=-\frac{K(t)}{\Pr (X^d_t=x)}, \end{aligned}$$

and the force is defined $F(x,t)\equiv -\partial _x V(x,t)$. While the strength of this force field varies spatially, its sign is the same for all *x* at a given time, meaning that the forces at every point are aligned in the same direction. This additional force corresponds to an addition to the drift velocity $v_\text {drift}'(x,t)=v_\text {drift}(x,t)+\Delta v_\text {drift}(x,t)$, given by:

$$\begin{aligned} \Delta v_\text {drift}(x,t)= \mu \Delta F(x,t), \end{aligned}$$

where the drift velocity is $v_\text {drift} \equiv \mu F(x,t)$. Finally, this adds to the probability current $J'(x,t)=J(x,t)+\Delta J(x,t)$. This turns out to be constant over position:

A2 $$\begin{aligned} \Delta J(x,t)&= \Pr (X_t^d=x) \Delta v_\text {drift}(x,t) \nonumber \\&= - \mu K(t). \end{aligned}$$

This constant probability current cannot be realized in an infinite positional variable since, despite locally preserving the probability distribution, probability flows out at one extreme end of the spatial degree of freedom.

To explicitly prove that an additional constant probability current is impossible in positional space ${\mathcal {X}}$ topologically conjugate to the real line ${\mathbb {R}}$, note that the Fokker–Planck equation Eq. (A1) is the continuity equation $\partial _t \Pr (X_t=x) = -\partial _x J(x,t)$. There is an integral form of this equation, which relates the change in probability in a region $[x_0,x_1]$ to the probability current through the boundary of the region:

$$\begin{aligned} \partial _t \int _{x_0}^{x_1} dx \Pr (X_t^d=x)= J(x_0,t)-J(x_1,t) . \end{aligned}$$

In order for $J'(x,t)$ to satisfy the Fokker–Planck equation, it must also satisfy $\partial _t \int _{x_0}^{x_1} dx \Pr (X_t^d=x)= J'(x_0,t)-J'(x_1,t)$. So far, there is no contradiction, since:

$$\begin{aligned} J'(x_0,t)-J'(x_1,t)&=J(x_0,t)- \mu K(t)-J(x_1,t)+ \mu K(t) \\&=J(x_0,t)-J(x_1,t). \end{aligned}$$

However, in the special case with $x_0=-\infty$—the region of interest is all $x \le x_1$—then the only boundary of the region is at $x_1$, such that:

$$\begin{aligned} \partial _t \int _{-\infty }^{x_1} dx \Pr (X_t^d=x)&=- J(x_1,t) \\&= -J'(x,t). \end{aligned}$$

For this to be true, *K*(*t*) must vanish, and so there cannot be any additional drift term. That is, up to an additional flat potential *C*(*t*), the counterdiabatic control protocols are the unique way to guide the system along a desired distribution trajectory $\{\Pr (X_t^d)\}$.

This proof does not preclude additional solutions with nonzero *K*(*t*) if the position variable has circular topology on a finite range $[x_0,x_1]$. This would mean that $x_0$ and $x_1$ are effectively adjacent such that there can be probability current at both points. In this case, there are always at least two boundary surfaces for any region, so it is impossible to use the integral continuity equation as above. The additional probability current *K*(*t*) is possible, due to probability flow between $x_0$ and $x_1$, which was not possible between $\infty$ and $-\infty$ in the previous case. However, this additional probability current corresponds to a force that points in the same direction along the loop, meaning that system is being driven cyclically. And so, the dynamics cannot be implemented with Hamiltonian control and must rely on some free energy resource to be sustained.

## Appendix B: Symmetric Metastable Erasure

In metastable erasure, we assume the system is in a metastable distribution of the initial symmetric equilibrium potential $V(x,0)=V(-x,0)$ during the entire protocol. If the two metastable informational states are $Y=0$, corresponding to $x \in (-\infty ,0]$, and $Y=1$, corresponding to $x \in (0,\infty )$, then we can describe a probability distribution trajectory as:

$$\begin{aligned} \Pr (X_t=x) = {\left\{ \begin{array}{ll} \Pr (X^\text {eq}=x)2\Pr (Y_t=0) &{} \text { if } x\le 0 \\ \Pr (X^\text {eq}=x)2\Pr (Y_t=1) &{} \text { if } x>0 \end{array}\right. }. \end{aligned}$$

We can then reparametrize in terms of the bit bias, that is the probability of the $Y=0$ informational state: $b(t)=\Pr (Y_{t}=0)$. As in Sec. 4.1, let $p(x) = \Pr (X^\text {eq}=x)$ denote the equilibrium distribution, which inherits the symmetry of the double well potential: $p(x) = p(-x)$. We then express the evolving metastable distribution as a function of control parameter:

$$\begin{aligned} \Pr (X_t=x) = {\left\{ \begin{array}{ll} 2p(x)b(t) &{} \text { if } x\le 0 \\ 2p(x)(1-b(t)) &{} \text { if } x>0 \end{array}\right. }. \end{aligned}$$

This expression allows us to simplify the counterdiabatic potential and counterdiabatic work, as follows.

$$\begin{aligned} V^{CD}(x,t)&= \frac{1}{\mu }\int _{0}^x dx' \frac{1}{\Pr (X_t=x')}\int _{-\infty }^{x'} dx'' \partial _t \Pr (X_t=x'') \\&={\left\{ \begin{array}{ll} \frac{1}{\mu } \int _{0}^x dx' \frac{1}{2bp(x')} \int _{-\infty }^{x'} dx'' 2p(x'') {\dot{b}} &{} \text { if } x\le 0 \\ \frac{1}{\mu }\int _{0}^x dx' \frac{1}{2(1-b)p(x')} (\int _{-\infty }^{0} dx''2 p(x''){\dot{b}} \int _{0}^{x'} dx'' 2p(x'')\partial _t (1-b)) &{} \text { if } x>0 \end{array}\right. } \\&={\left\{ \begin{array}{ll} \frac{1}{\mu }\int _{0}^x dx' \frac{1}{bp(x')} \int _{-\infty }^{x'} dx'' p(x''){\dot{b}} &{} \text { if } x\le 0 \\ \frac{1}{\mu }\int _{0}^x dx' \frac{1}{(1-b)p(x')} (\int _{-\infty }^{0} dx'' p(x''){\dot{b}} -\int _{0}^{x'} dx'' p(-x'') {\dot{b}}) &{} \text { if } x>0 \end{array}\right. } \\&={\left\{ \begin{array}{ll} \frac{1}{\mu }\int _{0}^x dx' \frac{1}{bp(x')} \int _{-\infty }^{x'} dx'' p(x''){\dot{b}} &{} \text { if } x\le 0 \\ \frac{1}{\mu }\int _{0}^x dx' \frac{1}{(1-b)p(x')} (\int _{-\infty }^{0} dx'' p(x''){\dot{b}} - \int _{-x'}^{0} dx'' p(x'') {\dot{b}}) &{} \text { if } x>0 \end{array}\right. } \\&={\left\{ \begin{array}{ll} \frac{1}{\mu }\int _{0}^x dx' \frac{1}{bp(x')} \int _{-\infty }^{x'} dx'' p(x''){\dot{b}} &{} \text { if } x\le 0 \\ \frac{1}{\mu }\int _{0}^x dx' \frac{1}{(1-b)p(x')} \int _{-\infty }^{-x'} dx'' p(x''){\dot{b}} &{} \text { if } x>0 \end{array}\right. } \\&={\left\{ \begin{array}{ll} \frac{1}{\mu }\int _{0}^x dx' \frac{1}{bp(x')} \int _{-\infty }^{-|x'|} dx'' p(x''){\dot{b}} &{} \text { if } x\le 0 \\ \frac{1}{\mu }\int _{0}^x dx' \frac{1}{(1-b)p(x')} \int _{-\infty }^{-|x'|} dx'' p(x''){\dot{b}} &{} \text { if } x>0 \end{array}\right. } \\&={\left\{ \begin{array}{ll} \frac{{\dot{b}}}{b}\frac{1}{\mu } \int _{0}^x dx' \frac{1}{p(x')} \int _{-\infty }^{-|x'|} dx'' p(x'') &{} \text { if } x \le 0 \\ \frac{{\dot{b}}}{1-b}\frac{1}{\mu } \int _{0}^x dx' \frac{1}{p(x')} \int _{-\infty }^{-|x'|} dx'' p(x'') &{} \text { if } x > 0 \end{array}\right. }, \end{aligned}$$

where $b=b(t)$ and $\dot{b} = \partial _t b(t)$. The second line follows from the first, since $p(x'')=p(-x'')$.

We can substitute $u=-x'$ again since $\int _{0}^x \frac{1}{p(x')}dx'=-\int _{0}^{-x} du \frac{1}{p(u)}$. And so, if we define:

$$\begin{aligned} h(x)= \frac{1}{\mu }\int _0^{|x|}dx' \frac{1}{p(x')} \int _{-\infty }^{-|x'|}dx''p(x''), \end{aligned}$$

then:

B1 $$\begin{aligned} V^{CD}(x,t)&= h(x) \times {\left\{ \begin{array}{ll} -\frac{{\dot{b}}}{b} &{} \text { if } x\le 0 \\ \frac{{\dot{b}}}{1-b} &{} \text { if } x>0 \end{array}\right. }. \end{aligned}$$

The resulting counterdiabatic work is:

$$\begin{aligned} \langle W^{CD} \rangle&= \int _{0}^{\tau } \!\! dt \int _{-\infty }^{\infty } \!\! dx \Pr (X_t=x) \partial _t V^\text {CD}(x,t) \\&=2 \int _{0}^{\tau } \!\! dt \int _{-\infty }^{0} \!\! dx \,p(x)b\,h(x) \partial _t \left( \frac{-{\dot{b}}}{b} \right) + 2\int _{0}^{\tau } \!\! dt \int _{0}^{\infty } \!\! dx \,p(x)(1-b)h(x) \partial _t \left( \frac{{\dot{b}}}{1-b} \right) \\&=2 \int _0^\infty \!\! dx \,p(x) h(x) \int _0^\tau \!\! dt \left( b \partial _t \left( \frac{-{\dot{b}}}{b} \right) +(1-b) \partial _t \left( \frac{{\dot{b}}}{1-b} \right) \right) \\&= 2 \int _0^\infty \!\! dx \,p(x) h(x) \int _0^\tau \!\! dt \left( -b \left( \frac{\partial _t^2 b}{b} -\frac{{\dot{b}}^2}{b^2}\right) +(1-b) \left( \frac{\partial _t^2 b}{1-b} +\frac{({\dot{b}})^2}{(1-b)^2} \right) \right) \\&=2 \int _0^\infty \!\! dx \,p(x) h(x) \int _0^\tau \!\! dt \left( -\partial _t^2 b +\frac{({\dot{b}})^2}{b}+\partial _t^2 b+\frac{({\dot{b}})^2}{(1-b)} \right) \\&= 2\int _0^\infty \!\! dx \,p(x) h(x) \int _0^\tau \!\! dt \left( \frac{({\dot{b}})^2}{b}+\frac{({\dot{b}})^2}{(1-b)} \right) \\&=2 \int _0^\infty \!\! dx \,p(x) h(x) \int _0^\tau \!\! dt \frac{({\dot{b}})^2}{b-b^2} \\&= f_1[p(\cdot )]\times f_2[b(\cdot )]. \end{aligned}$$

The second line follows from the first since *p*(*x*) and *h*(*x*) are symmetric. The functions appearing on the last line are given by:

$$\begin{aligned} f_2[b(\cdot )]=\int _0^\tau dt \frac{({\dot{b}})^2}{b-b^2} \end{aligned}$$

and:

$$\begin{aligned} f_1[p(\cdot )]&= 2\int _0^\infty dx \,p(x) h(x) \\&=2\int _0^\infty dx \,p(x) \frac{1}{\mu }\int _0^{|x|}dx' \frac{1}{p(x')} \int _{-\infty }^{-|x'|}dx''p(x'') \\&=\frac{2}{\mu } \int _0^\infty dx \,p(x) \int _0^{x}dx' \frac{1}{p(x')} \int _{-\infty }^{-x'}dx''p(x''). \end{aligned}$$

Thus, the counterdiabatic work is the product of two factors: one dependent on the bias trajectory *b*(*t*), containing all dependence on erasure fidelity, and the other dependent on the equilibrium potential *p*(*x*), containing all dependence on information storage robustness—the information lifetime.

One is tempted to use the functional $f_2$ to find the bias trajectory that minimizes dissipation. $f_2$ can be expressed as the integral of a Lagrangian:

$$\begin{aligned} f_2[b(\cdot )]=\int _{0}^{\tau }{\mathcal {L}}(b(t),b'(t)) dt , \end{aligned}$$

where:

$$\begin{aligned} {\mathcal {L}}(b(t),b'(t))=\frac{b'(t)^2}{b-b^2} . \end{aligned}$$

This implies that, for the optimal path satisfying the equation of motion:

B2 $$\begin{aligned} \frac{\partial {\mathcal {L}}}{\partial b}=\frac{d}{d t} \frac{\partial {\mathcal {L}}}{\partial b'}. \end{aligned}$$

Integrating these equations of motion, given the constraint of starting at initial bias $b(0)=b_i$ and ending at final bias $b(\tau )=b_f$, would determine the most thermodynamically efficient path *b*(*t*) for transiting between different metastable distributions. However, this is challenging and remains unsolved. So, instead, consider a simpler protocol.

We choose a bias trajectory:

$$\begin{aligned} b(t)=b(0)\cos (t \pi /2\tau )^2+b(\tau ) \sin (t \pi /2\tau )^2, \end{aligned}$$

with vanishing derivative at the protocol’s beginning and end, such that the desired distribution $\Pr (X^d_t)$ has zero time derivative at the initial and final times. This means that the counterdiabatic potential energy is zero at the protocol’s beginning and end. As a result, the system is in equilibrium at the beginning and end of the counterdiabatic step in the protocol.

Substituting this into the expression for $f_2[b(\cdot )]$ above, we evaluate numerically and see that the counterdiabatic work is proportional to the square of the Hellinger distance $K(\cdot ,\cdot )$:

$$\begin{aligned} f_2[b(\cdot )] = \frac{\pi ^2}{\tau }K^2(b(0),b(\tau )), \end{aligned}$$

where:

$$\begin{aligned} K^2(b,b') = \frac{ \left( \sqrt{b}-\sqrt{b'}\right) ^2+\left( \sqrt{1-b}-\sqrt{1-b'}\right) ^2}{2}. \end{aligned}$$

The proportionality’s form is forced since (i) the $\tau$ dependence factors out of the functional $f_2[b(\cdot )]$, as stated in the main text, and (ii) for the extreme bit-transfer case—$b(0)=0.0$ and $b(\tau )=1.0$—there is an analytic solution.

For bit transfer, the bias trajectory becomes $b(t)=\sin (t \pi /2 \tau )^2=(1-\cos (t \pi /\tau )) / 2$. This leads to an expression for the functional:

$$\begin{aligned} f_2[b(\cdot )]&= \int _{0}^\tau \frac{\left( \frac{\pi }{2 \tau } \sin (t \pi /\tau )\right) ^2}{\frac{1-\cos (t \pi /\tau )}{2}-\left( \frac{1-\cos (t \pi /\tau )}{2}\right) ^2}dt \\&= \frac{\pi ^2}{\tau } . \end{aligned}$$

Substituting our chosen bias trajectory into the expression for optimality in Eq. (B2), we see that it does not satisfy the equality, and so is not optimal.

## Footnotes

1 Symbolic dynamics is a long-lived subfield of dynamics systems [27]. Our use of it here is relatively simple, highlighting (i) how mesoscopic symbols capture (or not) collections of microscopic states and (ii) the concern of proper coarse-graining to locate information storage and processing. Fully deploying the symbolic dynamics for thermodynamic computing must wait for a different venue. Early results, however, do develop the symbolic dynamics of thermally-activated (noisy) systems [57].
